# A global map of the Zika virus phosphoproteome reveals host-driven regulation of viral particle release and cytopathogenicity

**DOI:** 10.1371/journal.ppat.1014300

**Published:** 2026-09-03

**Authors:** Inessa Manuelyan, Anna M. Schmoker, Boyd L. Yount, Philip Eisenhauer, Judith I. Keller, Clarissa Gold, Dante Terino, Christopher M. Ziegler, Jeff Alexander, Edward Hutchinson, David Bhella, Christopher D. Syme, Douglas G. Widman, Mark T. Heise, Ralph S. Baric, Bryan A. Ballif, Jason W. Botten

**Affiliations:** 1 Department of Medicine, Division of Immunobiology, University of Vermont Larner College of Medicine, Burlington, Vermont, United States of America; 2 Cellular, Molecular, and Biomedical Sciences Graduate Program, University of Vermont, Burlington, Vermont, United States of America; 3 Department of Biology, University of Vermont, Burlington, Vermont, United States of America; 4 Department of Epidemiology, The University of North Carolina at Chapel Hill Gillings School of Global Public Health, Chapel Hill, North Carolina, United States of America; 5 Vermont Biomedical Research Network, University of Vermont, Burlington, Vermont, United States of America; 6 VLP Therapeutics, Inc., Gaithersburg, Maryland, United States of America; 7 MRC-University of Glasgow Centre for Virus Research, University of Glasgow, Glasgow, Scotland, United Kingdom; 8 Department of Microbiology and Immunology, The University of North Carolina at Chapel Hill School of Medicine, Chapel Hill, North Carolina, United States of America; 9 Department of Genetics, The University of North Carolina at Chapel Hill School of Medicine, Chapel Hill, North Carolina, United States of America; 10 Department of Microbiology and Molecular Genetics, University of Vermont Larner College of Medicine, University of Vermont, Burlington, Vermont, United States of America; University of Massachusetts, Worcester, UNITED STATES OF AMERICA

## Abstract

Flaviviruses are enveloped, positive-strand RNA viruses that cause millions of infections in the human population annually. Although Zika virus (ZIKV) had been detected in humans as early as the 1950s, its reemergence in South America in 2015 resulted in a global health crisis. While flaviviruses encode 10 proteins that can be post-translationally modified by host enzymes, little is known regarding post-translational modifications (PTMs) of the flavivirus proteome. We used mass spectrometry to comprehensively identify host-driven PTMs on the ZIKV proteome. This approach allowed us to identify 43 PTMs across 8 ZIKV proteins, including several that are highly conserved within the *Flavivirus* genus. Notably, we found two phosphosites on the ZIKV envelope protein that are functionally important for viral propagation. Both appear to regulate viral particle release, while one also impacts ZIKV cytopathogenicity. Additionally, we discovered host kinases that interact with ZIKV proteins and determined that Bosutinib—an FDA-approved tyrosine kinase inhibitor that targets some of these host kinases—impairs ZIKV growth, in part by blocking phosphorylation of a tyrosine residue on the envelope protein. Thus, we have defined a high-resolution map of host-driven PTMs on ZIKV proteins as well as cellular interacting kinases, uncovered novel mechanisms of host driven-regulation of ZIKV particle release and cytopathogenicity, and identified an FDA-approved inhibitor of ZIKV growth.

## Author summary

Zika virus can cause neurological complications and birth defects in humans. The proteins made by viruses are the tools used to hijack human cells to repurpose them into virus production factories. We have a limited understanding of how the function(s) of these Zika virus proteins are regulated or the true range of functions each protein carries out. Herein, we created a comprehensive “map” of how human enzymes attach chemical tags—specifically phosphate and ubiquitin groups—to Zika virus proteins. These tags can act like molecular switches that can turn protein functions on or off. We discovered that two specific tagging sites on the virus’ outer shell protein, E, are critical for the efficient release of new virus particles from infected cells. Interestingly, blocking one of these tags also made the virus less harmful to cells, allowing the cells to survive longer. Furthermore, we identified an FDA-approved drug that successfully impairs virus growth, possibly by blocking these modifications. Our findings reveal new ways the virus controls its life cycle, a potentially novel role of the E protein for viral release, and suggest that repurposing drugs to target these host-driven processes could be a promising strategy for treating Zika virus infections.

## Introduction

**Fig 1:**
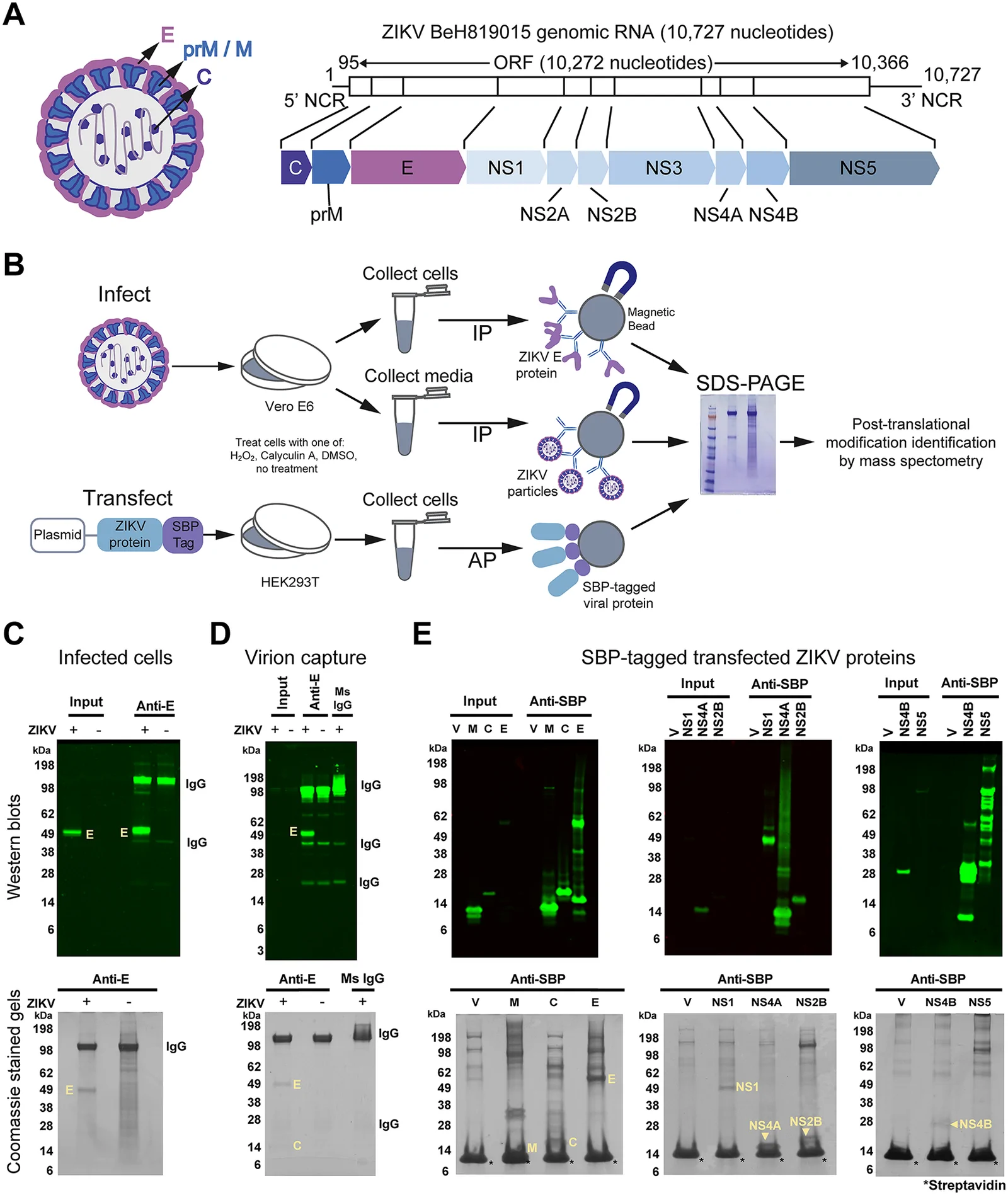
Discovery of post-translational modifications (PTMs) on the ZIKV proteome. (A) Depictions of i) a ZIKV particle with its structural proteins indicated and ii) the organization of the ZIKV positive-strand RNA genome and its encoded ORFeome. (B) Overview of the workflow for discovery of PTMs on ZIKV proteins in the settings of viral infection or plasmid-based expression of ZIKV proteins. IP, immunoprecipitation; AP, affinity purification. (C, D, and E) Western blots and Coomassie stained SDS-page gels of affinity purified ZIKV E from ZIKV infected cells (C), authentic ZIKV particles (D), or the indicated SBP-tagged ZIKV proteins (E). For (C and D), Vero E6 cells were infected (indicated by “+”) with ZIKV strain BeH819015 at an MOI of 0.1 or mock infected (indicated by “-”). Cells (C) and media (D) were collected 72 hours and 96 hours later, respectively. In (C), infected cells were lysed in Triton-X buffer and intracellular ZIKV E was purified using the anti-E antibody 4G2 and magnetic protein G beads. As a control, we performed immunoprecipitation of uninfected (mock) cellular lysates with the 4G2 antibody. In (D), viral particles were immunoprecipitated from clarified supernatants using the 4G2 antibody and magnetic protein G beads. Control conditions included (i) immunoprecipitation of virus-containing supernatants with a non-specific mouse IgG antibody and (ii) immunoprecipitation of an uninfected (mock) supernatant with the 4G2 antibody. For (E), to purify ZIKV proteins expressed from plasmid, we engineered plasmids to encode the indicated ZIKV ORFs with a C-terminal streptavidin-binding protein (SBP) tag. HEK293T cells were transfected with a plasmid encoding the indicated SBP-tagged ZIKV protein or, as a control, an empty vector (V). Two days after transfection, the cells were lysed in Triton-X buffer and ZIKV proteins were affinity purified using streptavidin-coated magnetic beads (labeled as Anti-SBP in panels). In panels (C-E), for both antibody- and streptavidin-based purifications, captured protein mixtures and corresponding control samples were orthogonally separated using SDS-PAGE, followed by in-gel digestion for subsequent mass spectrometry analysis to discover PTMs or interacting host proteins. The molecular weight in kDa, viral proteins (yellow text and arrows), and immunoglobulin bands are labeled. Note that the asterisks in (E) indicate monomeric streptavidin that was eluted off the streptavidin beads when boiled.

ZIKV is a mosquito-borne, enveloped, positive-strand RNA virus that encodes three structural proteins (capsid, C; membrane, prM; and envelope glycoprotein, E) and seven nonstructural (NS) proteins (NS1, NS2A, NS2B, NS3, NS4A, NS4B, and NS5; Fig 1A). ZIKV was first discovered in 1947 and confirmed as a human pathogen in 1952 [1]. ZIKV belongs to the *Flavivirus* genus, which has a large disease burden on the human population because of notable members like dengue (DENV), West Nile (WNV), and yellow fever viruses (YFV). Though considered a benign disease for most of its known history, ZIKV emerged in South America in 2015 and resulted in a global health crisis that lasted for more than a year and caused close to 1 million suspected and confirmed infections, but likely infected over 130 million people by the end of 2018 [2,3]. Most ZIKV infections are asymptomatic, while approximately 20% of those infected experience mild disease. In rarer cases, detrimental neurological complications arise in the form of Guillain-Barré syndrome as well as fetal microcephaly [4,5]. Infants afflicted with microcephaly because of ZIKV infection suffer developmental and health consequences into childhood often with no countermeasures available; those born with severe microcephaly require constant care.

Surveys of post-translational modifications (PTMs) on viral proteins have previously been used to understand how viruses affect cells and vice versa. Viral proteins are multifunctional and PTMs are an important host-driven process that can regulate viral protein functionality. Discovery of PTMs can potentially elucidate novel viral protein functions. For example, in human immunodeficiency virus and lymphocytic choriomeningitis virus (LCMV), phosphorylation of viral proteins has been shown to be important in key processes like viral particle budding and regulation of budding of different classes of viral particles [6–9]. There have been a small number of flavivirus phosphorylation sites discovered which demonstrate that phosphorylation of viral proteins can influence the host’s innate immune system or evade it, affect functionality of a viral protein, impact viral protein-protein interactions, or regulate the replication of viral RNA [10–14]. These important discoveries emerged from studies of NS5 proteins of several species of flaviviruses. However, there remains an expansive terrain of other flavivirus proteins. Importantly, identification of novel PTMs and their functional relevance is anticipated to reveal unexplored avenues for pharmacological therapeutics in these diseases. To address this gap in knowledge, herein we performed a large-scale mass spectrometry analysis of the ZIKV proteome to broadly map the PTM landscape of ZIKV. Additionally, we mapped host kinases that interact with the ZIKV proteome and tested whether FDA-approved inhibitors of interacting kinases can be used to inhibit ZIKV growth. Finally, we functionally interrogated a subset of the identified PTMs to determine their importance for ZIKV propagation and, where relevant, mapped the stage of the viral life cycle impacted.

## Results

### Discovery of post-translational modifications (PTMs) on the Zika virus proteome

A primary goal of this study was to identify host-driven PTMs that occur on ZIKV proteins produced by mammalian cells, either in the setting of cell-free virions or within cells. Where possible, we wished to examine PTMs in the setting of authentic ZIKV infection. At the time of this study, the only suitable antibody available for purification of a ZIKV protein, expressed in the context of an infected cell, was 4G2, which recognizes the ZIKV envelope (E) protein [15]. This antibody was used to purify intracellular ZIKV E and its interacting host partners from infected cells (Figs 1B and 1C) or E-decorated, cell-free virions released from these same infected cells (Figs 1B and 1D). Note that immunopurified ZIKV particles contained the structural proteins E, prM, and C (Fig 1D and S1 Data). We also isolated cell-free virions from infected mosquito cells via banding on a potassium tartrate density gradient (S1A Fig). To circumvent the lack of antibodies suitable for immunoprecipitation of the remaining ZIKV proteins from cells, we used plasmids to express streptavidin-binding protein (SBP)-tagged ZIKV proteins (Fig 1B). This allowed for efficient capture of SBP-tagged viral proteins via magnetic streptavidin beads (Figs 1E, S2, S3, and S4). To increase the likelihood of detecting phosphorylation events, transfected cells were treated with either H_2_O_2_, Calyculin A, or DMSO to inhibit tyrosine phosphatases, serine/threonine phosphatases, or to act as a control, respectively (Figs 1B, 1E, S2, S3, and S4). Samples derived from immunoprecipitation and affinity purification were subjected to SDS-PAGE to orthogonally separate protein components. Gel lanes were cut into multiple sections, and each piece was subjected to in-gel trypsin digestion. Meanwhile, ultracentrifuged purified virus from mosquito cells (S1B Fig) was digested with trypsin using filter-assisted sample preparation. All resulting tryptic peptides were subjected to liquid chromatography tandem mass spectrometry for identification of PTMs or interacting host proteins as described in Materials and Methods and previously [9,16]. A complete list of peptides from viral proteins including those containing PTMs such as phosphorylation and ubiquitination can be found in S2 Data. Tandem mass spectra for discovered phosphorylation and ubiquitination sites are available in S3 Data (spectra for ZIKV E T351 from virions is available in S1C, S1D Fig).

**Fig 2:**
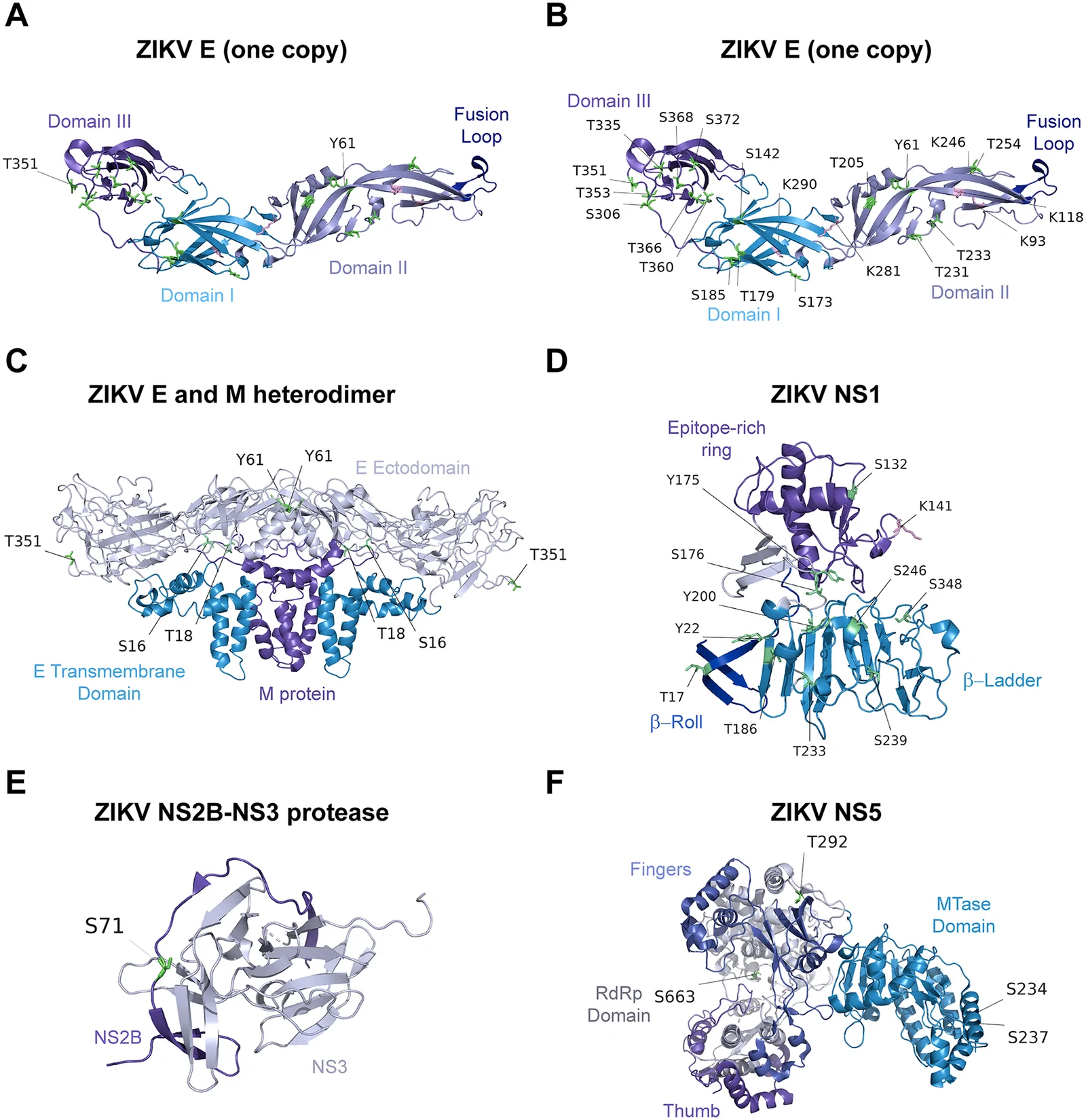
Location of post-translational modification sites on ZIKV proteins. Phosphosites and ubiquitination sites are marked in green and light purple, respectively. For each ZIKV protein, individual domains or specific features are color coded with the text label displayed in the matching color in each instance. (A and B) Location of the Y61 and T351 phosphosites on ZIKV E (A) or location of all PTMs on ZIKV E (B) with the exception of S149 and T156 which reside within an unresolved section of the structure. In both (A and B), PDB ID: 5JHM was used and one copy of E is shown. (C) ZIKV E - M heterodimer (PDB ID: 5IZ7). Y61 and T351 are labeled on the E ectodomain. ZIKV M phosphosites S16 and T18 are labeled on the M protein. (D) PTM locations on ZIKV NS1 (PDB ID: 5GS6). (E) ZIKV NS2B – NS3 protease complex shown in close conformation (PDB ID: 5GPI) with labeled phosphosites on ZIKV NS2B. (F) ZIKV NS5 (PDB ID: 5TMH) phosphosites. RdRp, RNA-dependent RNA polymerase; MTase, methyltransferase domain.

We identified 19 phosphorylation sites and 5 ubiquitination sites on ZIKV E (Tables 1, S1, Fig 2A, and 2B). Two of these phosphorylation sites, Y61 and T351, were discovered in the context of infection (Y61 from infected Vero E6 cells and T351 from mosquito cell-derived ZIKV particles) (S1D Fig). T351 was also found on ZIKV E expressed from plasmid in HEK293T cells. The remaining PTMs on E were identified from plasmid-expressed viral proteins (Table 1 indicates infection-derived sites). The locations of Y61, T351, and other PTMs found on ZIKV E are shown in Fig 2A and 2B. Y61 is located in Domain II of E, which is the region responsible for dimerization [17]. T351 is located in Domain III, which is often a target of neutralizing antibodies [17]. PTMs on E were also mapped using electrostatically labeled models (S5 Fig). S5A Fig shows one “face” of the ZIKV E dimer as largely positively charged while S5B Fig shows the other largely negatively charged face. In contrast, the border of the E dimer is mostly neutral (S5C Fig). The majority of ZIKV E phosphosites were found on the neutral border of the envelope protein (S5C Fig). The two phosphorylation sites on M, S16 and T18, are positioned in close proximity to one another at a junction of where E and M associate (Fig 2C). Interactions between the M S16 and T18 phosphorylated residues with E are hard to resolve, however, because both sites are in flexible regions of M and are interacting with flexible regions of E (S6A Fig).

**Table 1:** Phosphorylation and ubiquitination sites on the ZIKV proteome.

		Treatment^a^			
ZIKV Protein	Post-translational modifications^c^	DMSO	H_2_O_2_	Calyculin A	Predicted Kinases/Interacting kinases/ Closely-related kinases discovered^b^
**C**	None found				
**M**	S16ǂ^d^			✓	PRKAA1, NEK1, NEK3, NEK4, NEK5, NEK8, NEK10 (low), CLK2, **ATM**, **PRKDC** (min)
	T18			✓	**GSK3B**, AKT1, PRKACG (low), **CAMK2B**, PRKCE, PRKAA1, AURKA, **AURKB** (min)
**E**	**Y61**^^e^				FGR
	S142	✓	✓	✓	**GSK3B**, **AURKB**, NEK3 (min)
	S149	✓	✓	✓	PRKAA1, NEK5 (min)
	T156	✓			**GSK3B**, PLK1, PRKAA1 (min)
	S173^	✓	✓	✓	**CDK1**, **CDK5** (low), **MAPK3** (min)
	T179		✓	✓	PLK1, **PRKCD**, PRKCE, PRKAA1, AURKA, NEK6, **NEK7**, NEK10 (min)
	S185^ǂ^	✓		✓	
	T205			✓	**NEK7** (med), NEK6, NEK8, NEK9 (low), NEK 10 (min)
	T231	✓	✓	✓	NEK6, **MAPK3** (min)
	T233		✓	✓	**CDK1**, **CDK5** (low) **MAPK3** (low)
	T254			✓	PRKACG, **AURKB** (med), **PRKCD**, PRKAA1, AURKA (low), CAMK2G, PRKCE, PRKD1, NEK2, NEK4, NEK5 (min)
	S306^			✓	PLK1, AKT1, AURKA (min)
	T335	✓	✓	✓	CSNK1G2 (min)
	**T351** ^ǂ^	✓	✓	✓	CAMK2G (min)
	T353	✓	✓	✓	**CDK1**, **CDK5**, **MAPK3** (low)
	T360^^^f^			✓	AKT1 (low), PRKD1, **MAPK3** (min)
	T366			✓	
	S368			✓	
	S372			✓	NEK5 (low), CSNK1G2, PLK1 (min)
	K93^	✓	✓	✓	
	K118^		✓		
	K246	✓			
	**K281**^	✓	✓		
	K290	✓	✓		
**NS1**	T17^ǂ^*^g^	✓			PRKD1, PRKCZ (low) AKT1, PRKACG, PRKCA, PRKCD (min)
	Y22*	✓			FGR, LCK, SCR (min)
	S132	✓	✓	✓	**PLK1** (med), NEK5 (low), CAMK2G, PRKCD, PDPK1, NEK1, NEK5, NEK9, NEK10 (min)
	Y175		✓		INSR (min)
	S176	✓		✓	**PLK1** (low)
	T186			✓	CDK1 (min)
	Y200		✓		NEK10 (min)
	T233**^h^		✓		CSNK1G2 (min)
	S239**^^		✓		
	S246^			✓	PRKD1 (min)
	S348^^	✓	✓	✓	NEK2 (med), NEK1, NEK3, NEK4, NEK8, NEK10 (low), NEK5, NEK6, NEK9 (min)
	K141		✓		
**NS2A**	No expression				
**NS2B**	S71^		✓	✓	CDK1, CDK5 (low)
**NS3**	No expression				
**NS4A**	None found				
**NS4B**	S217^#i^/S218^#ǂ^/ T219^#^		✓		PRKAA1 (low), NEK 3, NEK6, NEK8, NEK9, GSK3B, PLK1, PDPK1, CDK1 (min)
**NS5**	S234			✓	CDK1 (min)
	S237^^			✓	PRKAA1, PRKDC, NEK3, NEK10 (min)
	T292^	✓			PRKCD, PRKCE, NEK6, NEK7, NEK8 (min)
	S663^^	✓	✓	✓	CLK2 (min)

^a^Cell treatment conditions (DMSO, H_2_O_2_, Calyculin A, or, in the case of Y61, no treatment) under which peptides were detected are indicated with check marks.

^b^Names in black text represent kinases predicted by Scansite 4.0 to phosphorylate the corresponding residue listed in a given row. Purple text represents predicted kinases that were interacting partners of the indicated viral protein for a particular row in this study. Bolded purple indicates that the kinase was independently discovered as an interacting partner of or functionally important for ZIKV in other studies. Blue text represents predicted kinases that were not found to interact with viral proteins but are in the same kinase cascade or have shared homology with an interacting kinase of the corresponding viral protein. Bolded black text represents predicted kinases that were an interacting partner with the viral protein in the corresponding row and/or were discovered in other ZIKV proteomics or functional studies, but were excluded after MiST analysis. Predicted kinases are followed by Scansite 4.0 stringency levels (med/medium, low, or min/minimum). Medium, low, minimum stringency indicates that the motif identified in the ZIKV polypeptide sequence is within the top 1%, 5% and 15% of sequence matches, respectively.

^c^Bolded text indicates phosphosites discovered in the context of ZIKV infection in this study (Y61, T351) or independently (K281) (29).

^d^**ǂ** Phosphosite conserved only among ZIKV strains.

^e^^ Phosphosite conserved in 7 or more of the 15 *Flavivirus* genus members analyzed.

^f^^^ Phosphosite highly conserved among 13 or more *Flavivirus* genus members analyzed.

-For full details of the amino acid conservation analysis related to ^d,e,f^, see the ‘multiple sequence alignment’ section of Materials and Methods and S4 Data.

^g*^Mass spectrometry data not sufficient to distinguish between NS1 sites T17 and Y22; there was evidence for both sites.

^h**^Evidence for both T233 and S239 phosphosites on NS1.

i#Phosphosite was located to one of these three locations.

We identified 11 phosphorylation sites and one ubiquitination site on NS1 (Tables 1, S1). Location mapping of PTMs on NS1 revealed that more than half of the modified amino acid residues were in the beta-roll or beta ladder domains of NS1 (Fig 2D) important for NS1 intracellular dimerization [18] and host protein interaction [19], respectively. We were unable to successfully express and isolate adequate quantities of NS2A or NS3 to analyze for PTMs (S7 Fig). Mass spectrometry revealed a phosphorylated serine at position 71 on the NS2B protein (Tables 1, S1), a small protein known to associate with NS3 to form the ZIKV protease complex (Fig 2E). There were no PTMs found on NS4A despite an abundance of NS4A peptides observed by mass spectrometry. One phosphorylation site was detected on NS4B, though the precise location could not be determined with certainty. Our mass spectrometry data suggest its location to be at either S217, S218, or T219 (Tables 1, S1). There are currently no models of ZIKV NS4B, thus we were unable to depict these sites within the protein structure. Lastly, we discovered four phosphorylation sites on NS5 (Tables 1, S1). Of these, two were located in the RNA-dependent RNA polymerase region of NS5 and two in the methyl-transferase region (Fig 2F).

To determine how conserved the discovered ZIKV protein PTMs might be with other flaviviruses, we constructed a multiple sequence alignment (MSA) that included viruses spanning the *Flavivirus* genus and chose flaviviruses known to cause human disease (S4 Data; virus list also noted in Materials and Methods). In total, we compared 15 different flaviviruses representing tick- or mosquito-borne viruses, viruses with no known vectors, and those considered emerging pathogens. Amino acid residues were defined as conserved if they were found in at least seven of the 15 flaviviruses in our panel. Of the 43 discovered PTMs, 14 met this degree of conservation across the *Flavivirus* genus and five were conserved across nearly all flavivirus subfamilies, a result that suggests they may be fundamentally important for many species of flaviviruses (Table 1). Notably, four sites were found only in ZIKV isolates (Table 1).

### Tyrosine 61 and threonine 351 on the ZIKV E protein are critical for viral growth

**Fig 3:**
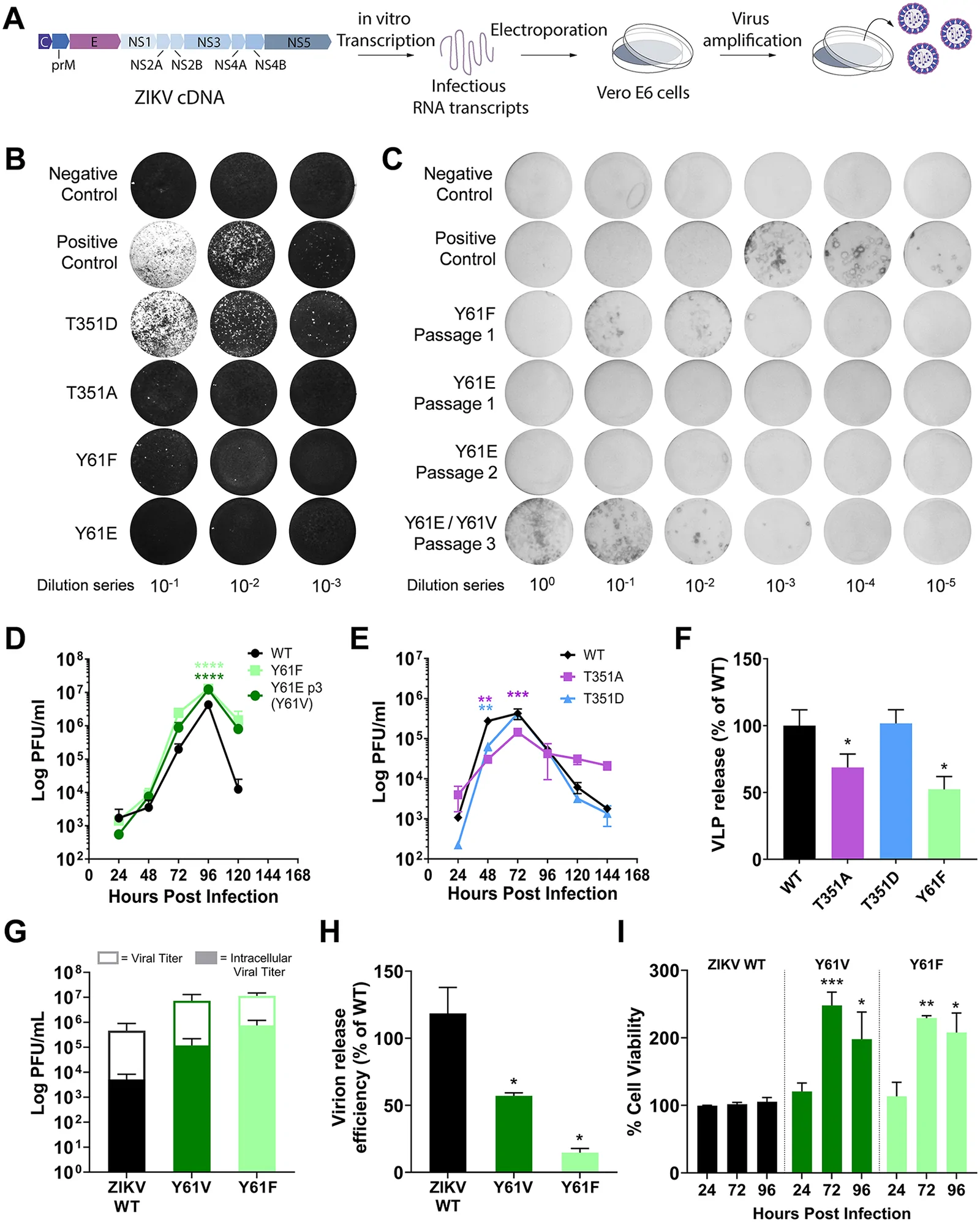
Tyrosine 61 and threonine 351 on the ZIKV E protein are critical for viral propagation at the stage of viral release. (A-C) Overview of the reverse genetics approach used to recover recombinant ZIKV bearing mutations at Y61 or T351 in the E protein (A). For each recovery attempt, full-length infectious RNAs were transcribed from a cDNA template of the viral genome. The RNAs were electroporated into Vero E6 cells and 3 days later supernatants from these cultures were screened for the presence of infectious ZIKV particles by either viral plaque assay (B) or focus assay (C); viral dilution series are indicated at the bottom of (B) and (C). In (C), the first three wells of the Positive Control dilution series (10^0^, 10^-1^, and 10^-2^) show no foci stain due to complete monolayer destruction and thus an absence of viral antigen to detect, while lack of staining in Negative Control wells is due to uninfected cells lacking viral antigen. The rY61E mutant virus was passaged two additional times due to the absence of detectable plaques or foci after passage 1. The rY61E passage 3 material showed infectious virus which had mutated from a glutamic acid to a valine at position 61 (rY61V). (D and E) The growth kinetics of the indicated rY61 mutants (D) or rT351 mutants (E) were determined by infecting Vero E6 cells at an MOI of 0.001 (D) or A549 cells at an MOI of 0.01 (E) with these viruses and measuring the infectious particles released by plaque assay at 24, 48, 72, 96, 120, or 144 hours after infection. Data represent the mean±SEM from three (D) or two (E) independent biological replicates. For statistical analysis, data were analyzed via mixed-effect analysis (two-way ANOVA) with Holm-Sidak’s test for multiple comparisons in Prism 10.4. (F) Virus-like particle (VLP) release was measured by expressing WT, Y61-, or T351-mutation containing E proteins. VLPs were generated by transfection of HEK293T cells with plasmid that encodes ZIKV prM and E (either WT E or the indicated E mutants). The quantity of ZIKV E released into the supernatant vs retained in cells was measured by quantitative western blot and VLP release efficiency was determined as described in Materials and Methods. Data represent the mean±SEM from three (rY61 mutants) or four (rT351 mutants) independent biological replicates. Statistical significance was determined in Prism 10.4 software using a one-way ANOVA with Holm-Sidak’s test for multiple comparisons. (G) The intracellular and extracellular titers of ZIKV rWT, rY61V, and rY61F were measured by plaque assay. Vero E6 cells were infected with the indicated virus at an MOI of 0.001 for 96 hours. Supernatants were collected, infected cells were lysed with ultrapure distilled water to release intracellular virions, as described in Materials and Methods, and titers were determined by plaque assay. Data represent the mean±SEM from two independent biological replicates. (H) Release efficiency of authentic virions was calculated by normalizing the ratio of rY61 mutant extracellular to intracellular infectious titers (shown in panel G) to the ratio of rWT extracellular to intracellular infectious titers, as described in Materials and Methods. Statistical significance was determined in Prism 10.4 software using a one-way ANOVA with Holm-Sidak’s test for multiple comparisons. (I) Cell viability after infection was measured by MTT assays. Vero E6 cells were infected with ZIKV rWT, rY61V, or rY61F virus at an MOI of 0.001 and cell viability was determined by MTT assay at 24, 72, and 96 hours post-infection, and normalized to the rWT condition. Data represent the mean±SEM from three biological replicates. Statistical significance was determined in Prism 10.4 software via two-way ANOVA with Holm-Sidak’s test for multiple comparisons. For the indicated statistical tests in (D-F): *, P < 0.05; **; P < 0.01; ***; P < 0.001; ****; P < 0.0001.

We next sought to determine whether the PTMs identified were important for viral propagation. We focused on two ZIKV E phosphosites, Y61 and T351, because they were found in both infection-derived and plasmid-expressed contexts. Y61 was discovered from infected cells while T351 was found in virions and on plasmid-expressed ZIKV E. Further, these sites represented residues that are either highly conserved in flaviviruses (Y61) or unique to ZIKV (T351) (Table 1; S4 Data). To evaluate their importance in viral propagation, we employed a ZIKV reverse genetics system [20] to generate recombinant viruses encoding phosphoablative or phosphomimetic mutations at these sites (Fig 3A). We then used these recombinant viruses in multi-step growth curves to determine how each change impacted viral fitness. Throughout this manuscript the terms “mutant(s)” and “mutant virus(es)” refer to recombinant infectious viruses carrying mutations at phosphorylation sites of interest that were recovered from a ZIKV infectious cDNA clone via reverse genetics. These mutants were compared to a recombinant wild-type Zika virus (rWT) rescued using the same reverse genetics system.

ZIKV E Y61 was mutated to a negatively-charged glutamic acid (Y61E) to reflect a permanent state of phosphorylation (i.e., a phosphomimetic mutant) and to a phenylalanine (Y61F) to represent a non-phosphorylatable state. We recovered the non-phosphorylatable Y61F mutant virus (rY61F) but, interestingly, the phosphomimetic Y61E mutant virus (rY61E) appeared to be nonviable as assessed by plaque assay (Fig 3B). Recovery of rY61E was attempted three times without success, despite successful generation of rWT each time. To account for the possibility that rY61E could be propagating but unable to form plaques (and therefore undetectable by plaque assay), we screened for the presence of infectious virus by focus assay (Fig 3C). In parallel, to account for the possibility that the mutant virus was replicating but not with sufficient kinetics to be detectable by focus or plaque assay after the initial recovery passage, we blind-passaged rY61E three times. This approach led to the successful isolation of a Y61E recombinant virus capable of forming foci beginning at passage 3 (Fig 3C). However, sequencing of the recombinant virus revealed that by passage 3, the glutamic acid had mutated to a valine (rY61V), suggesting that there is selection pressure to avoid a permanent negative charge at position 61.

To address whether phosphorylation of Y61 influences viral fitness, we performed a multi-step growth curve comparing the phosphomutants rY61F and rY61V with rWT. Interestingly, both rY61F and rY61V grew with accelerated kinetics and produced higher quantities of infectious virus compared to the wild-type (Fig 3D; p < 0.0001).

ZIKV E T351 was mutated to an aspartic acid (T351D) to mimic a state of phosphorylation or an alanine (T351A) to act as a non-phosphorylatable mutant. Recombinant viruses carrying these mutations were successfully recovered (Fig 3B) and multi-step growth curves were performed. There was no difference between the growth of the phosphomimetic rT351D and rWT at 72 hours, the peak of virus growth (Fig 3E; p = 0.968). Notably, at this same time point, the growth of the non-phosphorylatable rT351A was impaired compared to rWT or rT351D (Fig 3E; p < 0.001). After 72 hours, viral titers of rT351D and rWT decreased more rapidly compared to rT351A. This could be because rWT and rT351D caused more cytopathic effect at this time point and therefore cells could not support further rounds of infection.

In summary, both the ZIKV E Y61 and T351 sites appear important for viral propagation. Our results suggest that blocking phosphorylation at Y61 enhances viral growth while blocking phosphorylation at T351 impairs it.

### Y61 and T351 on ZIKV E are required for efficient Zika virus release

To determine the life cycle stage impacted by ZIKV E Y61 or T351, we next tested how a phosphomimetic or non-phosphorylatable mutation at each site would impact the release of viral particles from the cell. To do so, we used a virus-like particle (VLP) assay that features a single plasmid that expresses ZIKV prM and E. Once expressed, these two proteins form ZIKV VLPs that are secreted from cells and can be detected in the supernatant [21]. The plasmid was mutated at ZIKV E Y61 to Y61E or Y61F and at T351 to T351A or T351D. The Western blots used to quantify ZIKV E expression and calculate VLP release efficiency are shown in S8 Fig. Compared to the wild-type control, VLP release efficiency was decreased when T351 was mutated to alanine (T351A) but not aspartic acid (T351D) (Fig 3F; p = 0.049). Interestingly, we were unable to express ZIKV E protein when Y61 was mutated to Y61E (S8 Fig) despite performing two bacterial transformations and evaluating several clones from each transformation. This result, combined with our inability to recover genetically stable rY61E (Fig 3C), suggests that a constitutive negative charge or phosphorylation at Y61 is not favorable either for protein expression or viral growth. Mutation of Y61 to F also reduced VLP release efficiency (Fig 3F, p = 0.016), despite the improved growth kinetics of this mutant (Fig 3D).

We next measured authentic virion release efficiency of rY61F and rY61V by comparing the ratios of extracellular to intracellular infectious virions of the mutants to rWT. Notably, the intracellular infectious titers of both non-phosphorylatable mutants were higher than rWT (Fig 3G, S9A Fig: p = 0.0004 and p < 0.0001 for rY61V and Y61F, respectively), suggesting that intracellular virus particle budding was not negatively impacted. Yet, consistent with the VLP assay (Fig 3F), the efficiency of virion release from cells was decreased in both rY61V and rY61F (Fig 3H, p = 0.031 and 0.015, respectively; S9B Fig, p = 0.036 and p = 0.031, respectively).

Taken together, our findings indicate that phosphorylation at ZIKV E Y61 or T351 is required for efficient viral particle release.

### Phosphorylation status of ZIKV E Y61 impacts ZIKV cytopathogenicity

Titers of the non-phosphorylatable mutants rY61V and rY61F remained elevated at late time points (Fig 3D), leading us to assess whether phosphorylation at Y61 impacts cell viability. Using MTT assays, we measured the viability of Vero E6 cells infected with rWT, rY61V, or rY61F at 24, 72, and 96 hours post-infection (hpi). Compared to rWT, cells infected with either of the non-phosphorylatable mutant viruses showed increased viability at the later 72 and 96 hpi time points (Fig 3I; for rY61V at 72 and 96 hpi: p = 0.0008 and p = 0.011; for rY61F at 72 and 96 hpi; p = 0.0012 and p = 0.011). We verified that the viruses were adhering to the same growth kinetic patterns seen in Fig 3D by simultaneously measuring viral titers (S10 Fig). The highest cell viability, observed at 72 and 96 hours post-infection, matched the time points where rY61V and rY61F titers surpass rWT (S10 Fig). In summary, these results suggest that blocking host-driven phosphorylation of E Y61 protects cells from ZIKV cytopathogenicity.

### Host protein kinases interact with the Zika virus proteome

Given the multiple phosphosites discovered on various ZIKV proteins, we next wished to determine the host kinases that associate with ZIKV proteins. The immunoprecipitation and affinity purification performed in Figs 1C, 1D, 1E, S2, S3, and S4 not only captured ZIKV proteins, but also their host cellular protein partners. Thus, we were able to identify host kinases that interacted with ZIKV C, M, E, NS1, NS2B, NS4A, and NS4B. In total, we found 115 cellular kinases that interacted with ZIKV proteins (S2 Table). We subjected this dataset to analysis using MiST, an affinity purification mass spectrometry computational tool that scores protein-protein interactions to remove interactions that could be false positives [22]. MiST analysis restricted the interactome to 40 kinases (S3 Table, S11 Fig) that were above a MiST threshold of 0.62. This threshold was chosen based on how inclusive it was of kinases that were independently discovered in other ZIKV proteomic or functional screens [23–28] but still stringent enough to remove low-scoring interactions. Most kinases were unique to this study, but 14 were independently discovered in other ZIKV proteomic or functional screens (S3 Table, Column F). Notably, when comparing the original list of 115 kinases to these studies [23–28], an additional 18 kinases overlap with our study (S2 Table, Column F). We have included the complete list of 115 kinases in S2 Table in the event that MiST analysis excluded additional, not-yet-tested, but functionally relevant interactions. S11 Fig depicts the MiST analyzed kinases and highlights their involvement in biological processes such as regulation of protein translation, neuron differentiation, cell cycle arrest, and other cellular processes which are relevant to ZIKV infections.

**Fig 4:**
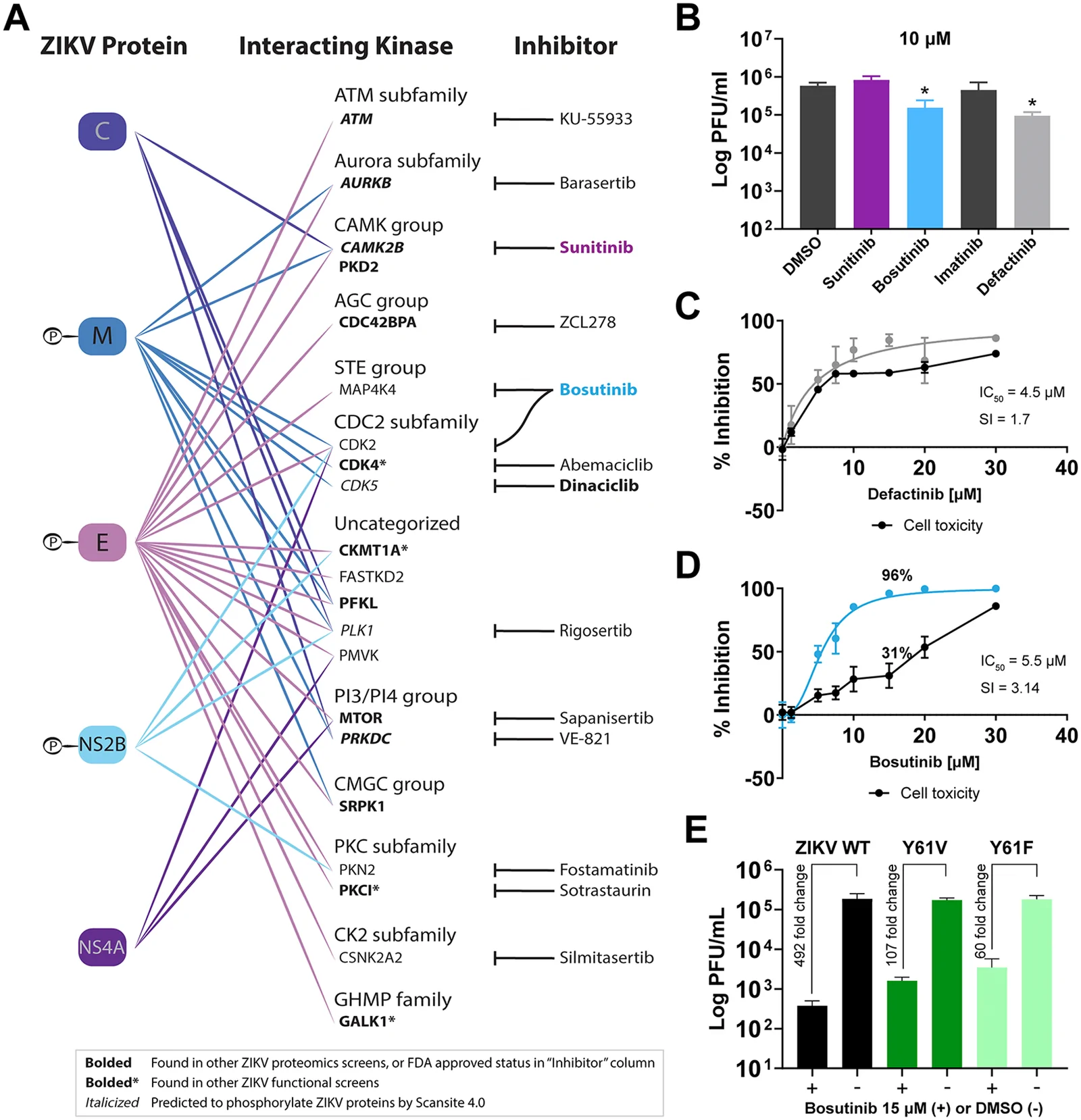
Host kinase interactions the ZIKV proteome and effects of kinase inhibitors on ZIKV growth. (A) Host kinases that interact with ZIKV proteins representative of MiST-scored interactions with a chosen threshold of >0.8. In the “Interacting Kinase” column, kinases are organized into respective groups or families. Bolded text indicates kinases that were also found to interact with ZIKV proteins in other studies. Bolded text with an asterisk were found to be functionally important in other studies. Kinases predicted by Scansite 4.0 to phosphorylate ZIKV proteins at the discovered phosphosites are italicized. To capture the range of the different kinases found, we prioritized displaying kinases discovered in published ZIKV interactomes or functional studies, then those that had the highest MiST scores. Drugs known to inhibit specific kinases are listed in the “Inhibitor” column; drugs with FDA approval are bolded. Note the full list of interacting kinases are listed in S2 Table. (B) Efficacy of kinase inhibitors in restricting ZIKV growth. A549 cells were infected with ZIKV at a MOI of 0.1. One hour later, the inoculum was removed and cells were treated with 10 µM of the indicated drug or, as a control, DMSO (vehicle). Two days later infectious virus was quantified from supernatants via plaque assay. Data represent mean±SEM from three independent biological replicates. Statistical significance was determined in Prism 10.4 software via one-way ANOVA with Holm-Sidak’s test for multiple comparisons. *, P < 0.05. (C and D) IC_50_ determination for Defactinib and Bosutinib inhibition of ZIKV growth. A549 cells were infected with ZIKV at a MOI of 0.1 and 1 hour later, the inoculum was removed and cells were treated with the indicated doses of Bosutinib, Defactinib, or as a control, DMSO (vehicle). Two days later infectious virus was measured in the supernatants via plaque assay. In parallel, the cytotoxicity of each drug at these same doses was determined via MTT assay as described in Materials and Methods. Data represent mean±SEM from three independent biological replicates. The resulting IC_50_ and selective index (SI) values are listed for each drug in the Results section. Calculations of both values are described in Materials and Methods. (E) Bosutinib inhibits growth of non-phosphorylatable rY61 mutant viruses, but to a lesser degree than rWT virus. A549 cells were infected with ZIKV rWT, rY61V, or rY61F at an MOI of 0.01. One hour post-infection, inoculum was removed and replaced with cell media containing either Bosutinib [15 µM] or DMSO. Supernatants were collected after 48 hours, and titers were measured via plaque assay. Data represent the mean±SEM from three biological replicates. Statistical analysis of the differences in fold changes between groups was determined in Prism 10.4 software via one-way ANOVA with Holm-Sidak’s test for multiple comparisons and showed no statistical difference.

We next used Scansite 4.0 to predict kinases that might phosphorylate ZIKV proteins at the sites of phosphorylation identified in this study (Tables 1, S2 and S3, Column I). Intriguingly, a subset of these kinases were interacting partners of the ZIKV proteins they were predicted to phosphorylate and/or were discovered in other ZIKV proteomic or functional screens (Table 1, purple text if unique to our dataset, or bolded purple text if discovered in other ZIKV studies). These were AURKB, CAM2KB, PLK1, and PRKDC (serine/threonine-protein kinase Aurora B, calcium/calmodulin-dependent protein kinase type II subunit beta, serine/threonine-protein kinase 13, and DNA-dependent protein kinase catalytic subunit, respectively). This list is more extensive when not accounting for MiST scoring (Table 1, bolded black text). Other kinases predicted by Scansite 4.0, although not found by our mass spectrometry analysis to be interacting partners, either have shared homology with or are found in the same signaling cascades as interacting kinases (Table 1, highlighted in blue). To incorporate the information above, we constructed Fig 4A which is representative of kinase interactions with MiST scores above 0.8 and highlights the broad range of kinases detected from various kinase groups or families/subfamilies, emphasizing those which were discovered in other ZIKV proteomic (bolded text), functional screens (bolded and asterisked text), and predicted by Scansite 4.0 to phosphorylate a ZIKV protein (italicized text). Fig 4A also includes inhibitors known to target the listed kinases, some of which are FDA-approved (bolded text in “Inhibitor” column). In summary, we have identified ZIKV-interacting host kinases, some of which were predicted to phosphorylate ZIKV proteins at sites discovered in this study, and a number of which that were previously confirmed as ZIKV interactors or important for ZIKV propagation (Tables 1, S2, S3, Fig 4A).

### Inhibition of host cell kinases by Bosutinib impairs Zika virus growth and its mechanism of action is partially associated with blocking Y61 phosphorylation on ZIKV E

Considering that there are no FDA approved treatments for ZIKV, we next tested whether selected FDA-approved kinase inhibitors might be effective in restricting ZIKV growth. At 10 µM, the tyrosine kinase inhibitors Bosutinib and Defactinib both reduced ZIKV titers (Fig 4B; p = 0.03, p = 0.02, respectively) while Sunitinib and Imatinib did not (Fig 4B; p = 0.17, p = 0.35, respectively). To determine the IC_50_ of each drug, we constructed concentration-response curves. To account for drug cytotoxicity, we performed simultaneous MTT assays on cells that had been treated with each kinase inhibitor at the equivalent concentrations. Although Defactinib inhibition of ZIKV infectious virus production reached levels as high as 86%, at the same concentration of drug, cell toxicity measured 73% (Fig 4C). At Defactinib’s IC_50_ of 4.5 µM, cell toxicity was approximately 46%. The fitted curve of inhibition and cell toxicity closely followed each other, suggesting that most of the viral inhibition may have been due to nonviable cells. After determining the CC_50_ of Defactinib to be 7.53 µM, we calculated the selectivity index for Defactinib to be 1.7. The IC_50_ of Bosutinib was 5.5 µM. In this case, at the same doses of drug, cell toxicity was approximately 17%. At higher concentrations, Bosutinib completely inhibited viral growth while cell toxicity remained at approximately 30% (Fig 4D). Given Bosutinib’s IC_50_ and CC_50_ (17.43 µM), we calculated the selectivity index to be 3.14. Because Bosutinib is a tyrosine kinase inhibitor, we next wanted to determine if ZIKV impairment by the drug was associated with blocking phosphorylation of Y61, the only phosphotyrosine discovered on the ZIKV E protein. We thus tested whether Bosutinib would impair rY61V and rY61F growth despite their inability to be phosphorylated. We found that 15 µM of Bosutinib, a concentration chosen based on its full inhibition of WT virus (Fig 4D), indeed reduced rY61V and rY61F titers by 107- and 60-fold, respectively (Fig 4E). However, the titer reduction was not as robust compared to the 492-fold change in rWT. Thus, Bosutinib appears to inhibit ZIKV partially by its inhibitory effect on a host kinase or kinases responsible for Y61 phosphorylation, but also through other avenues such as blocking phosphorylation on a different ZIKV tyrosine (i.e., NS1 Y22, Y175, Y200, or one that was not detected in this study) and/or by effecting host proteins that lead to cellular events which are unfavorable for ZIKV growth. In summary, we and others have shown that ZIKV proteins interact with host kinases and some of these host kinases are predicted to phosphorylate ZIKV proteins at the discovered phosphosites in this study (Tables 1, S2, S3). Finally, we have shown that the mechanism by which Bosutinib reduces ZIKV growth (Fig 4D) may be due, in part, to blocking phosphorylation of Y61. Overall, our results suggest that host cell kinases may be tractable antiviral targets for ZIKV.

## Discussion

Little is known regarding the diversity of host-driven PTMs that occur on flavivirus proteins or how such modifications regulate the functionality of these proteins. We addressed this deficiency by providing the first comprehensive map of phosphorylation and ubiquitination sites found on a flavivirus proteome. A major finding was that selected phosphorylation sites on the ZIKV E protein appear critical for ZIKV propagation, likely at the stage of viral particle release. Moreover, we discovered that blocking phosphorylation of a tyrosine on ZIKV E reduced ZIKV cytopathogenicity, leading to a paradoxical increase in infectious virus, despite a defect in viral release. We also identified a network of host kinases that interact with ZIKV proteins, some of which were predicted to target the identified phosphosites. Finally, we discovered that Bosutinib, an FDA approved tyrosine kinase inhibitor, likely impairs ZIKV growth partly by blocking phosphorylation of a newly discovered ZIKV E phosphotyrosine. Our data suggest that inhibition of host kinases may be an effective approach to restrict ZIKV growth.

The use of mass spectrometry allowed us to discover 43 sites of phosphorylation and ubiquitination on ZIKV proteins. Due to the lack of antibodies suitable for immunoprecipitation, many of these PTMs were discovered on plasmid-expressed viral proteins in non-infectious settings, introducing a limitation to this study. PTMs identified under these conditions may not fully reflect those present during authentic ZIKV infection and should be confirmed in the context of infection in future studies. Our subsequent experiments prioritized sites with infection-derived evidence: ZIKV E phosphosite Y61 from infected cells and T351 from authentic virions as well as from plasmid-expressed ZIKV E. The functional importance of T351 suggests that PTMs identified on plasmid-expressed viral proteins can be functionally relevant. In another example, two ubiquitination sites on ZIKV E were shown to be required for viral entry and replication [29] and one of these K281, was detected on plasmid-expressed ZIKV E in our study (Tables 1, S1), highlighting the value of plasmid-expressed viral protein PTMs for guiding follow-up functional studies.

The process of flavivirus particle formation and release from the host cell begins with virus particle assembly and budding into the endoplasmic reticulum. These immature particles then traffic to the Golgi and move through the trans-Golgi network (TGN), eventually being released into the extracellular environment after fusion of a TGN transport vesicle with the plasma membrane [30]. We show that the ZIKV envelope glycoprotein (ZIKV E) has at least two phosphosites, Y61 and T351, which are involved in the viral egress process, with Y61 acting specifically at the release stage (Figs 3F, 3H, S9B). Thus, our data suggest that phosphorylation of E could play a regulatory role in ZIKV release. To our knowledge, phosphorylation of a flavivirus protein has not been implicated in the release process. Moreover, considering the conservation of Y61 in other pathogenic flaviviruses (Table 1, S4 Data), this PTM could be fundamentally important for flavivirus release.

Phosphosite Y61 is found in domain II of ZIKV E, the domain responsible for ZIKV E dimerization [17]. The inability of the phosphomimetic mutant virus rY61E to efficiently grow over the first two passages following reverse genetics rescue coupled with its conversion at this residue from a glutamic acid to valine by passage 3 (Fig 3C, 3D) suggest that the negatively charged amino acid is not favorable. Indeed, there may be pressure at position 61 to be either a non-charged or hydrophobic amino acid. A zoomed-in view of the ZIKV model reveals that Y61 is near an asparagine (N207) and glutamic acid (E262) (S6B Fig). In the absence of phosphorylation, it is possible that the N207 and E262 residues are interacting. A large phosphate group on Y61 might repel the negative charge on E262 and perhaps disrupt this interaction, providing a possible explanation for why a non-charged state is optimal at this position. This preference is further supported by the fact that we readily rescued the non-phosphorylatable mutant, rY61F, which has a hydrophobic, non-charged phenylalanine at position 61.

The hydrophobic/non-phosphorylatable amino acid changes (Y to F or V) at position 61 resulted in recombinant ZIKV mutants that were less cytopathic (Fig 3I) and outperformed recombinant wild-type ZIKV in reaching peak titers (Fig 3D, 3G), yet resulted in a release defect (Figs 3F, 3H, S9B). We interpret these findings in several ways. First, phosphorylation of ZIKV E at Y61 is either not favorable to the virus or is beneficial at very specific time point(s) in the ZIKV life cycle, supported by the observation of the release defect. If the latter scenario is at work, our growth curve data may not be sufficient to resolve at which other life cycle stages Y61 phosphorylation may be beneficial to ZIKV because the recombinant viruses employed contained static mutations at this position. The negatively-charged phosphosite may be important in aiding a specific purpose, such as encouraging a conformational change for a period and then returning to an unphosphorylated state.

When mutated to Y61E, ZIKV E no longer expressed from a plasmid (S8 Fig), suggesting that the ZIKV envelope protein containing a negatively-charged glutamic acid at this position is too unstable for expression or may be quickly marked for degradation. Interestingly, the Y61 site is highly conserved among flaviviruses. A multiple sequence alignment (S4 Data) shows that flaviviruses such as WNV, YFV, Japanese encephalitis virus (JEV), and Saint Louis encephalitis virus (SLEV) also contain a tyrosine at this position, suggesting that maintaining control over the charge of this tyrosine likely benefits these flaviviruses. DENV contains an isoleucine at this position and more distantly related flaviviruses contain a leucine, both of which share properties with tyrosine and phenylalanine in that they are also non-charged, hydrophobic amino acids.

**Fig 5:**
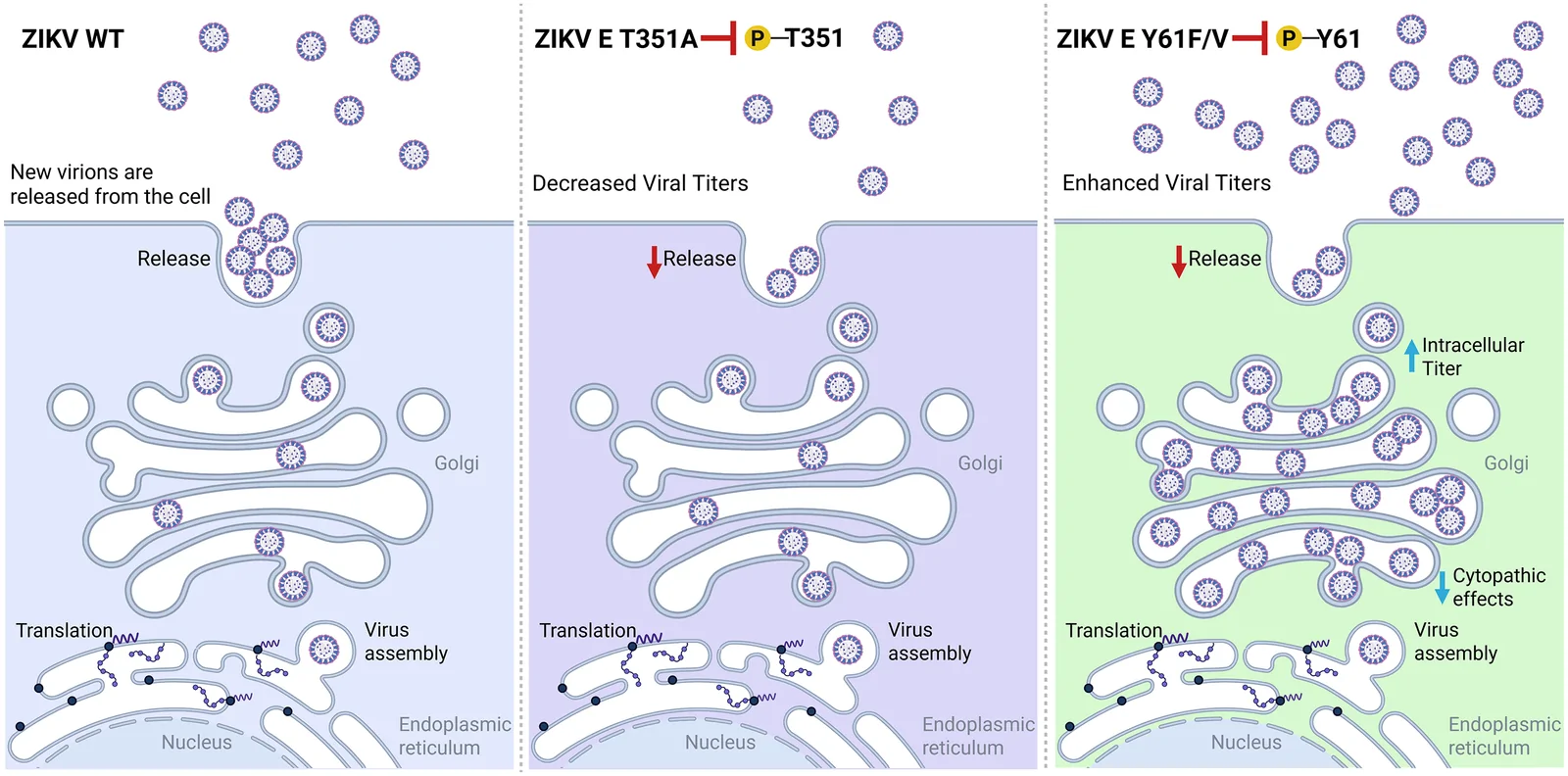
Graphical summary of the effects of blocking phosphorylation of ZIKV E T351 and ZIKV E Y61 on ZIKV growth. A graphical interpretation of results from Figs 3D–3I and S9 experiments. Blocking phosphorylation of ZIKV E T351 reduces viral release efficiency, resulting in a viral titer decrease. Blocking phosphorylation of ZIKV E Y61 reduces viral release efficiency, increases intracellular viral titers, and reduces ZIKV cytopathogenicity. Presumably, the decrease in virus-induced cytopathogenicity supersedes the negative impact on virus release, resulting in an overall higher capacity for viral propagation. Created in BioRender. Manuelyan, I. (2026) https://BioRender.com/vfnqbs4 [50].

The seemingly paradoxical characteristics of decreased VLP or authentic virion release (Figs 3F, 3H, S9B) but enhanced growth of the rY61 mutants (Fig 3D) are resolved when considering their significantly dampened cytopathogenicity (Fig 3I). With increased cell viability, the mutant viruses may be replicating to higher levels, evidenced by the increased intracellular titers of rY61V and rY61F compared to rWT (Figs 3G, S9A), overcoming their release defects and ultimately outperforming rWT (summarized in Fig 5).

Phosphorylation of ZIKV E at position T351 was observed both in purified virions and in cells. T351 sits within Domain III of ZIKV E, which contains motifs for cellular receptor binding and is often the target of neutralizing antibodies [17]. A negative charge at this position, either from a stochastically phosphorylated threonine on the wild-type virus (rWT) or an aspartic acid substitution on the rT351D mutant virus, was optimal for viral growth (Fig 3E). Similarly, a negatively charged aspartic acid on ZIKV E (T351D) resulted in equivalent levels of VLP release as WT. Conversely, the non-phosphorylatable alanine mutant, T351A, resulted in a strong reduction in VLP release (Fig 3F), suggesting that host-driven phosphorylation of T351 promotes ZIKV particle release. Intriguingly, phosphorylation of T351 was discovered both on ZIKV E isolated from human cells as well as on virions derived from mosquito cells, suggesting that not only is phosphorylation of T351 important in the host, but that it may also play a role in the virus vector. Future studies could focus on confirming the functional importance of T351 and other discovered phosphosites, such as Y61, in mosquito cells, as well as evaluating whether phosphomutant viruses from mosquito cells impact entry or infectivity in human cells. Because of differences in human and arthropod kinomes, it is possible that the ZIKV phosphoproteome in vector cells would diverge from our dataset in mammalian cells. Additionally, it may be that optimal viral fitness requires different patterns of regulatory phosphorylation in the cells of mammalian and arthropod hosts. Therefore, it would be informational to map the phosphoproteome of ZIKV in mosquito cells for comparison with this dataset as well as for comparison of the roles of phosphorylation in both cell types.

Previous studies have shown that host-driven phosphorylation of the flavivirus NS5 protein is critical for the flavivirus viral life cycle. For example, phosphorylation of YFV NS5 at a highly conserved site (S56) is required for YFV to evade the cap-dependent innate immune response in the host cell [10]. Blocking the phosphorylation of another conserved flavivirus residue, T449 on DENV NS5, results in a nonfunctional protein [31]. Other key studies linked phosphorylation of NS5 or Hepatitis C virus NS5A to important functions such as its interaction with NS3 [11,32], localization during different stages of infection [32], and viral particle assembly and viral replication [12,33]. In some instances, phosphorylation of NS5 was detected but not the specific residues driving the observed phenotype [11,32]. The highly conserved NS5 phosphosites identified in our study may be responsible for driving these important functions (Table 1). Indeed, we have uncovered several highly conserved phosphosites in the ZIKV proteome that may help guide the discovery of new functions for these viral proteins or the mechanisms by which these functions are regulated.

Our mass spectrometry analysis revealed 115 host kinases that interacted with one or more of the 8 ZIKV proteins probed (S2 Table). A limitation of this study is that we did not orthogonally verify these interactions. An additional consideration is that kinase expression in the cell lines used may not fully reflect that of primary cells targeted by ZIKV, which could influence both the phosphoproteomic landscape and the observed kinase and viral protein interactions. Therefore, it will be important to validate interactions and functionally interrogate these kinases in future studies. However, despite these potential limitations, 32 of the kinases discovered in this study were identified in other ZIKV proteomics or functional studies, providing independent validation of our findings. We used MiST analysis to refine the list of kinases from 115 to 40 and remove potential false positives (S3 Table). This resulted in the exclusion of 18 independently discovered kinases [23–28], introducing the possibility that the analysis might remove functionally relevant targets. Therefore, in addition to the abridged MiST-selected kinases listed in S3 Table and S11 Fig, we have also provided the complete list of 115 interacting kinases in S2 Table.

The discovery of host kinases as viral protein interactors raised the possibility that some subset may be responsible for viral protein phosphorylation. Treating cells after infection with Bosutinib, an FDA-approved tyrosine kinase inhibitor, reduced ZIKV growth (Fig 4B, 4D). Indeed, a recent study by Valencia *et al.* confirmed our finding that Bosutinib reduces ZIKV growth in the setting of BHK-21 cells [34]. There are different possibilities for how Bosutinib inhibits ZIKV growth. One is that Bosutinib’s inhibitory effect on ZIKV is exclusively through cellular mechanisms. For example, Bosutinib may be acting on host kinases that ultimately lead to a cellular environment not conducive to optimal ZIKV growth. Another possibility is that Bosutinib reduces titers by inhibiting kinases that act directly on ZIKV phosphosites and cellular processes do not play a role in titer reduction. However, Bosutinib is known to act on many cellular kinases [35], making it unlikely that the reduction of ZIKV titers is solely due to its impact on viral phosphosites. Which leads to the last possibility that Bosutinib may be acting through a combination of cellular mechanisms as well as directly on ZIKV phosphosites. To investigate whether Bosutinib’s mechanism of action is associated with blocking phosphorylation of Y61, the lone high confidence phosphotyrosine discovered on ZIKV E, we treated the rY61V and rY61F mutant viruses with Bosutinib. If Bosutinib’s only mechanism for reducing ZIKV titers is by blocking Y61 phosphorylation due to the inhibition of a kinase that acts on this site, and the mutant viruses are refractory to this kinase because they are non-phosphorylatable, then we would expect that Bosutinib treatment would not lower mutant virus titers. Conversely, if Bosutinib reduces ZIKV titers through mechanism(s) completely independent to blocking Y61 phosphorylation, then mutations at this site would be irrelevant and we would expect Bosutinib to reduce mutant virus titers as effectively as it does ZIKV WT titers. Our results showed that Bosutinib reduced the mutant virus’ titers, but to a lesser degree than rWT (Fig 4E), suggesting that Bosutinib’s titer reduction is, in part, due to blocking phosphorylation at Y61 and/or potentially different ZIKV phosphosite(s) (i.e., NS1 Y22, Y175, Y200, or one not detected in this study) and/or by acting on sites of tyrosine phosphorylation on key cellular targets. It is possible that Bosutinib treatment, by blocking Y61 phosphorylation, results in decreased particle release, similar to the decreased release of VLPs (Fig 3F) and authentic virions (Figs 3H, S9B) of the non-phosphorylatable rY61F and rY61V mutants. In the mutant viruses, this release defect was ultimately overcome by higher intracellular (Figs 3G, S9A) and extracellular (Figs 3D and 3G, S9A) titers stemming from a substantial increase in cell viability (Fig 3I).

Although the kinase predicted by Scansite 4.0 to phosphorylate Y61, FGR, was not detected as a ZIKV interacting partner, the closely related tyrosine protein kinase FYN was (Tables 1, S2). Furthermore, FYN is inhibited by Bosutinib at nanomolar concentrations [35]. Additional studies are needed to identify the exact host kinase that phosphorylates Y61. As discussed above, Bosutinib may be affecting other ZIKV phosphosites and we have found additional kinases that are targeted by the drug. It is also important to consider that the concentration at which kinase inhibitors are administered will influence their specificity. So, although targets of Bosutinib (Fig 4A) were not predicted to directly phosphorylate discovered phosphosites (Table 1), Bosutinib may be inhibiting off-target kinases that phosphorylate ZIKV proteins. Further studies using molecules related to Bosutinib may improve efficacy and specificity for blocking specific kinases. Collectively, these data imply that blocking phosphorylation by host kinases is a potential antiviral strategy.

Many of our discovered kinases have been corroborated as ZIKV interactors or functionally important for ZIKV (Fig 4A, Tables 1, S2, S3) [23–28]. Of these, several (ATM, AURKB, CAMK2B, GSK3B, NEK7, PRKDC) are predicted to phosphorylate ZIKV at the phosphosites discovered in this study, making them intriguing targets for further studies (Table 1). Several interacting kinases (ATM, ATR, and mTOR) discovered in our study and others (S2 Table) [25,26] were the targets of early drug screens against ZIKV. Specifically, Cherry *et al*. used inhibitors VE-822 and WAY-600 to robustly block ZIKV infection [36]. Intriguingly, VE-822 targets kinases ATM and ATR, which we found to be interacting partners of ZIKV M (ATM, ATR) or ZIKV E (ATM) (note that ATM is predicted to phosphorylate S16 on ZIKV M; Table 1). WAY-600 targets mTOR, which we found to be an interacting partner of ZIKV phosphoproteins M and E as well as NS4A (S2 and S3 Tables). Interestingly, mTOR was the most abundant kinase found to interact with any of the ZIKV proteins (S2 Table, Column H). Its importance was established early in the ZIKV epidemic when it was discovered that ZIKV proteins can suppress the Akt-mTOR pathway, leading to impairment of neurogenesis and upregulation of autophagy, which is beneficial for ZIKV during replication [37,38]. Another study found an overall downregulation of the AKT/mTOR pathway during ZIKV infection [25]. Due to ZIKV M associating with high levels of mTOR (see spectral counts in S2 Table, Column H), we theorize that ZIKV M could be acting as a sink for mTOR, providing an explanation for why the Akt-mTOR pathway is downregulated and there is less phosphorylation of mTOR targets observed during ZIKV infection [25].

In summary, we have detailed a high-resolution map of host-driven PTMs on ZIKV proteins, uncovered a novel mechanism by which ZIKV release may be regulated, discovered that phosphorylation may influence ZIKV cytopathogenicity, and identified an FDA-approved drug that could be repurposed for the treatment of ZIKV infection. The dataset described here will be useful to the field for the discovery of novel ZIKV protein functions and the mechanisms by which these functions are regulated. Our work further suggests that targeting host kinases may be an effective antiviral strategy and provides potential targets.

## Materials and methods

### Cells and viruses

HEK 293T/17 cells (CRL-11268), referred to as HEK293T throughout, and A549 cells (CCL-185) were procured from the American Type Culture Collection (ATCC, Manassas, VA). Vero E6 cells were kindly given by J. L. Whitton (The Scripps Research Institute, La Jolla, CA). HEK293T cells were maintained in Dulbecco’s modified Eagle medium (DMEM) containing 10% fetal bovine serum (FBS), 1% penicillin-streptomycin, 1% HEPES buffer solution, 1% MEM nonessential amino acid solution, and 1% GlutaMAX, from Thermo Fisher Scientific (Waltham, MA). A549 cells were maintained in DMEM-F12 (Thermo Fisher Scientific) containing 10% FBS and 1% penicillin-streptomycin. Vero E6 cells were maintained in DMEM containing 10% FBS, 1% penicillin-streptomycin, and 1% HEPES buffer solution (Thermo Fisher Scientific). Cell lines described above were incubated at 37°C with 5% CO_2_. *Aedes albopictus* mosquito cells (C6/36; a kind gift of Prof Alain Kohl, MRC-University of Glasgow Centre for Virus Research) were maintained in Leibovitz’s L15 media (Gibco) containing 10% FBS (Gibco), 10% triptose phosphate broth (Gibco), and 1% pen/strep (Gibco) and incubated at 28^o^C. ZIKV strain BeH819015, a Brazilian isolate (GenBank: KU365778.1), was generously provided by the Baric Lab (University of North Carolina, Chapel Hill, North Carolina, USA) [20]. The Zika virus strain used to infect mosquito cells was ZIKV/H.sapiens/Brazil/PE243/2015 (Recife, Brazil) [39]. Working stocks of ZIKV, including recombinant ZIKV generated via reverse genetics (see below for details on recovery), were grown on Vero E6 cells and titered by standard plaque assay.

### Plasmids

Several plasmids were used for expression of ZIKV proteins. The nucleotide sequences of ZIKV ORFs used in this study were derived from ZIKV strain H/PF/2013 (NCBI gene identifier number AHZ13508). The Heise lab (University of North Carolina, Chapel Hill, North Carolina, USA) provided their pOME plasmid-based library that permits expression of each ZIKV ORF containing a C-terminal 3XFLAG tag [40]. To enable streptavidin-based affinity purification of ZIKV proteins, the ZIKV C, E, M, NS1, NS2B, and NS4A ORFs encoded in the pOME plasmids were subcloned into our modified pCAGGS expression vector [41,42] such that they would encode a C-terminal streptavidin binding peptide (SBP) (MDEKTTGWRGGHVVEGLAGELEQLRARLEHHPQGQREP) in place of the 3XFLAG tag. Note that we expressed the mature sequences of NS4A and NS4B (omitting the 2K peptide) to identify host-interactors or PTMs of the final, proteolytically processed forms of these proteins. Each ZIKV ORF was fused to the SBP tag through an 18 base pair linker. The nucleotide sequence of the ZIKV genes was amplified by PCR from the pOME vectors with forward primers that contained a 5’ overhang containing a Gateway AttB1 site and a Kozak sequence and reverse primers containing an overhang with the linker sequence (all primers used are listed in S4 Table). The SBP tag was amplified from a previously generated in-house plasmid via PCR using the forward primer SBP (5’- GCAGCTGGAGGTGGAGGTATGGACGAAAAAACCACCGGT-3’), which has a 5’ overhang containing the linker sequence, and the reverse primer SBP (5’-ACCACTTTGTACAAGAAAGCTGGGTCTTACGGTTCACGCTGACCCTGCGG-3’), which contains a 3’ overhang with a stop codon preceding an AttB2 sequence. The two PCR products (each respective amplified ZIKV gene and the amplified SBP tag) were fused by PCR using the appropriate ZIKV gene forward primer and the SBP reverse primer. The resulting cassette was subcloned into the modified pCAGGS vector using the Gateway cloning system (Invitrogen) following the manufacturer’s instructions. pOME plasmids encoding ZIKV NS2A, NS3, NS4B, or NS5, each with the same C-terminal linker and SBP tag as described directly above, were generated by GenScript Biotech Corporation (Piscataway, NJ, USA) but were maintained in the original pOME vector. Plasmids for the ZIKV reverse genetics system were kindly provide by the Baric Lab as described in Widman *et al.* [20]. The plasmid from the Baric system which contained the ZIKV E gene was modified by GenScript to include the Y61F, Y61E, T351A, and T351D mutations. The plasmid used for the VLP release assay, which encodes the ZIKV prM and E genes, is described here [21] and was generously provided by Emergent BioSolutions, Inc. This plasmid was also modified by GenScript to include the Y61F, Y61E, T351A, and T351D mutations. All plasmid sequences were verified by DNA sequencing.

### Identification of post-translational modifications of ZIKV proteins by mass spectrometry

To identify phosphorylation sites on ZIKV proteins via mass spectrometry, three general approaches were taken. One biological replicate was performed for each approach. In the first approach, to purify ZIKV E, Vero E6 cells were infected with ZIKV at an MOI of 0.1 or mock infected and 72 hours later cells were collected and lysed in Triton buffer consisting of 1% Triton X-100, 0.5% NP40, 140mM NaCl, and 25mM Tris-HCl containing a protease inhibitor cocktail (04693159001, Roche Applied Science, Indianapolis, IN) as well as PhosStop phosphatase inhibitor cocktail (04906837001, Roche Applied Science). The resulting protein lysate was incubated with anti-ZIKV E 4G2 antibody at 4°C, overnight. The next day, magnetic Protein G Dynabeads (Thermo Fisher Scientific) were added to the lysate-antibody mixture and incubated at 4°C, rotating for at least 4 hours (but not more than 8 hr). Beads were then magnetically isolated, gently washed 3 times in Triton buffer, magnetically isolated for a final time and then boiled in Laemmli buffer (62.5 mM Tris-HCl, 10% glycerol, 2% sodium dodecyl sulfate and 0.01% bromophenol blue (B392, Fisher Scientific, Pittsburgh, PA) for 5 minutes to release antibody, ZIKV E protein, and associated host or viral protein partners. Similarly, ZIKV virions were immunopurified from clarified infected Vero E6 supernatants 96 hours post-infection using Dynabeads coated with anti-ZIKV E 4G2 antibody or an irrelevant mouse IgG then lysed using 10X Triton buffer containing the same protease and phosphatase inhibitor cocktails described directly above. The mock infected cell lysates and supernatants served as controls during mass spectrometry analysis.

To purify ZIKV proteins expressed from plasmid, our second approach was to transfect HEK293T cells with plasmid expressing ZIKV C, M, E, NS1, NS2B, NS4A, NS4B, or NS5 proteins with a C-terminal SBP tag in each case, or an empty plasmid labeled as V for “vector” in Figs 1E, S2, S3, S4 to serve as the control condition during mass spectrometry analysis. 5x10^5^ cells per well in 6-well plates were transfected with 2 μg of plasmid using 8 μg of polyethylenimine (PEI, 23966, Polysciences, Inc., Warrington, PA). Two days later, transfected cells were treated either with DMSO (D2650, Sigma-Aldrich. Saint Louis, MO), H_2_O_2_, (Thermo Fisher) or Calyculin A (Cell Signaling Technology, Danver, MA) for 20 minutes. Cells were collected and lysed with Triton buffer (same formulation described above) and then subjected to affinity purification using magnetic MyOne Streptavidin T1 beads (65601, Thermo Fisher Scientific) according to the manufacturer’s instructions. Briefly, magnetic beads were washed in Triton buffer several times, added to protein lysates from transfected cells, and then rotated overnight at 4°C. The next day, the beads were magnetically isolated and washed several times with Triton buffer containing protease and phosphatase inhibitors and then boiled in Laemmli buffer for 5 minutes to release SBP-tagged ZIKV proteins from the streptavidin beads.

Infected or mock infected cell lysates (control condition), transfected lysates from cells expressing ZIKV proteins or empty plasmid/empty vector (control condition), and virion protein lysates from infected or mock infected supernatants (control condition) were separated on 4–12% Bis-Tris polyacrylamide gels (NW04120BOX, Thermo Fisher Scientific). To visualize protein bands for excising and mass spectrometry processing, gels were stained with Coomassie (40% methanol, 20% acetic acid, and 0.1% Brilliant Blue R (B7920, Sigma-Aldrich)). Gels were destained with a solution of 30% methanol and 10% acetic acid and imaged using a Canon Canoscan 8800F scanner. Sample lanes were cut into a series of gel pieces containing prominent protein bands or band groups. After excision, gel pieces were further cut into 1 mm cubes and processed. Chemicals used for processing were purchased from Thermo Fisher Scientific. Gel pieces were rinsed with HPLC-grade water and then incubated with destain solution (50 mM ammonium bicarbonate and 50% acetonitrile) for 30 minutes at 37°C. Destain was removed and gel pieces were dehydrated by incubating twice with 100% acetonitrile for 5 minutes. The gel pieces were reduced with 25 mM dithiothreitol in 50 mM ammonium bicarbonate for 30 minutes at 55°C. After cooling, gel pieces were dehydrated with 100% acetonitrile for 5 minutes and then alkylated with 10 mM iodoacetamide in 50 mM ammonium bicarbonate for 45 minutes at room temperature, while protected from light. Gel pieces were washed twice in destain solution for 5 minutes, dehydrated with 100% acetonitrile, then rehydrated with water for 10 minutes. Gel pieces were further dehydrated with two 5-minute incubations in 100% acetonitrile. All liquid was removed and gel pieces were left to incubate at room temperature to evaporate trace acetonitrile. Gel pieces were rehydrated with a solution of 12.5 ng/μL sequencing-grade, modified trypsin (V5111, Promega) in 50 mM ammonium bicarbonate on ice for 30 minutes, before digesting overnight at 37°C. Peptides were extracted with a solution of 2.5% formic acid in 50% acetonitrile while spinning in a microcentrifuge at 13,000 RPM for 10 minutes. The supernatant was removed and saved while the gel pieces were subjected to further extraction and rinsing with 100% acetonitrile. The second extraction was combined with the initial extraction. All solvent was removed from the extracts using a vacuum centrifuge at 37°C. The peptides were resuspended in 2.5% formic acid, 2.5% acetonitrile prior to mass spectrometry analysis.

Dried peptides were resuspended in Solvent A (2.5% MeCN, 0.15% formic acid (FA)) and separated using the Easy n-LC 1200 across 15-cm columns packed in-house with 2.7 µm C18 packing material prior to analysis on the Q Exactive Plus mass spectrometer fitted with a Nanospray Flex ion source (2.2 kV) and supplied with Thermo Xcalibur 4.0 software. Peptides were eluted using a 0–50% gradient of Solvent B (80% MeCN, 0.15% FA) over 60 min, followed by 10 minutes at 95% Solvent B. The precursor scan (scan range = 360–1700 *m/z*, resolution = 7.0 x 10^4^, maximum IT = 100 ms) was followed by HCD fragmentation spectra in a Top-10 approach (resolution = 3.5 x 10^4^, AGC = 5.0 x 10^4^, maximum IT = 50 ms, isolation window = 61.6 *m/z*, normalized collision energy = 26%, dynamic exclusion = 30 s). Raw spectra were searched against a forward and reverse database of tagged ZIKV proteins with common contaminants added using SEQUEST with a precursor mass tolerance of ± 5 PPM and a fragment ion tolerance of ± 0.006 Da. The following differential modifications were permitted: phosphorylation of serine, threonine and tyrosine (±79.9663 Da), ubiquitylation of lysine (±114.0429 Da), oxidation of methionine (±15.9949 Da), carboxyamidomethylation of cysteine (±57.0215 Da), and acrylamidation of cysteine (±71.0371 Da). No enzyme was indicated in the original search to expand the database, and the resulting hits were filtered for tryptic peptides. Spectra of phosphopeptide hits were manually assessed and compared to their unphosphorylated counterparts to confirm hits and determine phosphorylation site localization. Any phosphopeptide hits with poor fragmentation or with ambiguous phosphorylation site localization were removed from the dataset (S2 Data). To identify bound human interactors, raw data were searched against a forward and reverse human database containing common contaminants via SEQUEST. Trypsin was specified as the cleavage agent with two missed cleavages permitted. Mass tolerances and allowed modifications were as stated above. Resulting hits were filtered by cross correlation score (>= 2.0 for z = 2, >= 2.2 for z = 3, >=2.4 for z = 4, >=2.6 for z = 5) and delta correlation score (>=0.15), resulting in an FDR of <1%. Host proteins were considered ZIKV interactors if they were identified by two or more unique peptides in experimental conditions and were not present in the controls. Proteins were also considered ZIKV interactors in cases where proteins were >5-fold enriched in experimental over control conditions. We used the MiST computational tool at https://modbase.compbio.ucsf.edu/mist in the PCA (principal component analysis) training mode to score the viral protein – host protein interactions identified by mass spectrometry. The MiST “reproducibility” feature was weighted at 0, as interactor data were from one biological replicate; the final MiST score was a composite of weighted “abundance” and “specificity” scores. After scoring, a threshold of 0.62 was chosen to generate S3 Table and S11 Fig. Fig 4A included kinases with scores greater than 0.8.

In our third approach, which led to the identification of ZIKV E T351 from virions, C6/36 cells were infected with ZIKV at an MOI of 0.1 and then incubated at 28^o^C. Four days later, the supernatant was harvested and clarified by centrifugation at 5000 RPM. The clarified supernatant was concentrated using 100,000 MW cut-off Centricon concentrators (Millipore). The concentrate was underlayed with a 5 mL sucrose cushion (24% w/v in 1x NTE buffer; NaCl-Tris-EDTA) and ultracentrifuged at 105,000 *g* in a Surespin 630 Rotor (Thermo Fisher Scientific) for 1.5 hours. The pellet-associated virus was resuspended in NTE, then overlaid onto a 10–35% (w/v) / density gradient of potassium tartrate in 30% glycerol in NTE, prepared using a Gradient Master (Biocomp Instruments). Virions were banded by ultracentrifugation at 175,000 *g* overnight and collected by pipetting from the top of the gradient. Next, the virus-containing fraction was removed from the gradient, placed in 100,000 MW cut-off dialysis units, and dialyzed several times using 1 × NTE to remove the potassium tartrate and glycerol. After dialyzing overnight, virus was concentrated using 100,000 MW cut-off Centricon concentrators (Millipore).

Concentrated ZIKV virions were inactivated at 70°C in 4M final urea in TEAB and then prepared for liquid chromatography and tandem mass spectrometry (LC-MS/MS) as described [43]. Briefly, the sample was reduced, alkylated, and then digested with trypsin and LysC using filter-assisted sample preparation (FASP) [44]. Peptides were separated by reversed-phase chromatography using a 2h gradient on an Ultimate 3000 RSLCnano HPLC system (Dionex). This was run in direct injection mode and coupled to a Q Exactive mass spectrometer (Thermo Fisher Scientific), running in ‘Top 10′ data-dependent acquisition mode with fragmentation by higher-energy collisional dissociation (HCD). Charge state + 1 ions were rejected and fragmentation and dynamic exclusion with 40 s was enabled. Mass spectra were annotated in MaxQuant version 1.6.3.4 [45] with reference proteomes from Zika virus isolate ZIKV/H.sapiens/Brazil/PE243/2015 (GenBank KX197192.1) and *Aedes aegypti* (UniProt UP000008820, accessed on 2018-10-26) and the MaxQuant common contaminants list. Standard settings were used, with the enzyme set as trypsin/P and fixed modifications set as carbamidomethyl (C). Variable modifications were oxidation (M), acetyl (Protein N-ter), phospho (STY) and, to account for artefactual modifications when inactivating in urea, carbamyl (KRC). Modified spectra were considered where they could be identified in the absence of artefactual carbamylation. Data from this third approach can be found at https://researchdata.gla.ac.uk/1222/.

### Multiple sequence alignment

A multiple sequence alignment (MSA) of flavivirus FASTA-formatted sequences was generated using the MUSCLE (Multiple Sequence Comparison by Log-Expectation) algorithm [46] as implemented in the NIAID Virus Pathogen Database and Analysis Resource (ViPR) through the web site at http://www.viprbrc.org/ [47]. The Uclust parameter was set as a MUSCLE pre-processor to improve both speed and quality of alignment. The alignment visualization with PTM annotations was created in Jalview 2.11.1.4 [48]. The following flaviviruses were aligned: ZIKV H/PF/2013 (AHZ13508), ZIKV Uganda (ABI54475), ZIKV SPH2015 (ALU33341), ZIKV Paraiba (ANH10698), ZIKV USA UT (AOO19564), ZIKV BeH819015 (AMA12085), DENV 1 NP059433), DENV 2 (NP056776), DENV 3 (YP001621843), DENV 4 (NP073286), JEV (Japanese encephalitis virus; NP059434), SLEV (Saint Louis Encephalitis Virus; YP001008348), USUV (Usutu virus; AAS59401), WNV 1(YP001527877), WNV 2 (NP041724), AHFV (Alkhurma hemorrhagic fever virus; AAL08421), KFDV (Kyasanur forest disease virus, AAQ91607), OHFV (Omsk hemorrhagic fever virus, AAQ91606), TBEV (tick-borne encephalitis virus; NP043135), ENTV (Entebbe bat virus; AAV34153), MMLV (Montana myotis leukoencephalitis virus, CAC82713), LAMV (Lammi virus, ACR56717), YFV (NP041726), and PCV (Palm Creek virus, AGG76014).

### SDS-PAGE and western blotting

Protein lysates were diluted in Laemmli buffer, boiled for 5 minutes, then separated on NuPAGE 4–12% Bis-Tris gels with MES buffer. Protein was transferred to nitrocellulose membranes using iBlot gel transfer stacks (IB301001 or IB301002, Invitrogen) and the Invitrogen iBlot Device according to manufacturer instructions. Membranes were blocked with 5% milk in PBS for 1 hour and incubated overnight with primary antibody diluted in PBS which contained 5% milk and 0.2% Tween 20 (BP337, Thermo Fisher Scientific). The following day the membrane was rinsed several times with TBST (tris-buffered saline with 0.1% Tween 20) then incubated for 1 hour with secondary antibody diluted in PBS containing 5% milk, 0.2% Tween 20, and 0.02% SDS. After secondary antibody incubation, the membrane was washed several times in TBST then washed in PBS before imaging on a LI-COR Odyssey CLx system.

Primary antibodies were used for western blotting at the following concentrations: mouse anti-ZIKV E (4G2) (1:10,000) (generously provided by Baric Lab), mouse anti-streptavidin binding peptide (MAB10764, Millipore, Billerica, MA) (1:10,000), rabbit anti-actin (A2066, Sigma-Aldrich) (1:5,000), and mouse anti-actin (A5441, Sigma-Aldrich) (1:5,000). For quantitative western blotting, the following secondary antibodies from LI-COR were used at a 1:20,000 dilution: IRDye 800CW goat anti-mouse (926–32210) and IRDye 680LT goat anti-rabbit (926–68021).

### Generation of recombinant ZIKV

We used the Baric Lab reverse genetics system to generate phosphomimetic and non-phosphorylatable mutants as described in Widman *et al.* [20]. ZIKV E was mutated at Y61 to Y61E and Y61F and separately ZIKV E T351 was mutated to T351D and T351A. The reverse genetics system is composed of 4 plasmids, containing Fragments A through D, that encode the ZIKV proteome. Fragment A was modified by GenScript from thymine to guanine at nucleotide position 1599 and thymine to adenine at position 1601 to generate the Y61E phosphomimetic while nucleotide 1600 was modified from adenine to thymine for the Y61F non-phosphorylatable mutant. Nucleotides 2470 and 2471 in Fragment A were modified from adenine and cytosine to guanine and adenine, respectively, for the T351D phosphomimetic while position 2470 was modified from adenine to guanine for the T351A non-phosphorylatable mutant. Recombinant viruses were generated as described in Widman *et al.* [20]. The four plasmids were digested with BamHI and Bsu36I for Fragment A, Bsu36I and BstXI for Fragment B, BstXI and SfiI for Fragment C, and SfiI, SmaI, AlwNI for Fragment D. Restriction enzymes were all acquired from New England Biolabs (Ipswich, MA). The four digested fragments were ligated with T4 DNA ligase (New England Biolabs). Ligated DNA was precipitated with chloroform then *in vitro* transcribed using the mMESSAGE mMACHINE T7 ULTRA Transcription Kit (Thermo Fisher Scientific). The transcribed product was then electroporated into Vero E6 cells using a Gene Pulser Xcell (Bio-Rad Laboratories, Hercules, CA) and incubated at 37°C for 4 days. Cell media was collected after 4 days and passaged onto a new monolayer of Vero E6 cells. Cell media collected from this passage was described as “passage 1/ p.1”. Clarified cell media was titered by plaque assay using Vero E6 cells.

### Virus growth curves

For the rY61F and rY61E mutant viruses, six-well plates were seeded with 2 x 10^5^ Vero E6 cells per well and infected the following day at an MOI of 0.001. For the rT351A and rT351D mutant viruses, 12-well plates were seeded with 2 x 10^4^ A549 cells and infected the following day at an MOI of 0.01. Cell media was collected at indicated time points from separate wells. Cell media was clarified by centrifugation at 1200 RPM for 5 minutes, stored at -80°C, and then titered by plaque assay (see plaque and focus assays below for details).

### Plaque and focus assays

Plaque assays were used to measure infectious virus titers in several experiments as follows. Six-well plates were seeded with 2.5 x 10^5^ Vero E6 cells per well and the following day inoculated with 10-fold serial dilutions of virus. Plates were rocked every 15 minutes for 1 hour to allow for viral absorption at 37°C, then cells were overlaid with a solution of 0.7% agarose (20–102, Apex Industrial Chemicals, Aberdeen, United Kingdom) in Vero E6 growth media. Wells with agarose plugs were fixed 3 days later with a solution of 25% formaldehyde (1635-4L, Sigma-Aldrich) in 3x PBS. Following removal of the agarose, the fixed cell monolayers were stained with 0.1% crystal violet (C581-100, Thermo Fisher Scientific) and 2.1% ethanol in water.

Focus assays were used to assess the presence of rY61E after several viral passages in Fig 3C and for measuring infectious virus titers of experiments in S9A Fig. 24-well or 96-well plates were seeded with 2 x 10^4^ or 1 x 10^4^ Vero E6 cells per well, respectively, and the following day were inoculated with 10-fold serial dilutions of recombinant viruses. Plates were incubated for 1 hour at 37°C with rocking every 15 minutes. After absorption, cells were overlaid with 0.01% methylcellulose in Vero E6 media (see Cells and Viruses for media detail) and were left in a 37°C incubator for 3 days or 2 days (for 96-well plates). After, the overlay was removed and cell monolayers were fixed with 4% formaldehyde (28906, Thermo Fisher Scientific) at room temperature, washed, then permeabilized with 0.5% Triton X-100 in PBS. Cell monolayers were then blocked with 5% nonfat milk (dry powder, Thermo Fisher Scientific) in PBS and thereafter incubated with 4G2 antibody (1:10,000) diluted in 5% nonfat milk in PBS for 1 hour at 37°C. Cells were washed with PBS and then incubated with goat anti-mouse secondary antibody (5450–0011, LGC Clinical Diagnostics, Inc., Milford, MA) at a 1:2000 dilution in 5% nonfat milk in PBS for 1 hour at 37°C. Cells were washed again and then incubated with TrueBlue Peroxidase Substrate (5510–0030, LGC Clinical Diagnostics, Inc.) until the formation of blue foci and reaction was stopped by removing the substrate.

### Virus-like particle (VLP) release assay

To determine the efficiency of VLP formation and release when ZIKV E contains mutations at Y61 or T351, 5 x 10^5^ HEK293T cells in 6-well plates were transfected with 2 µg of the modified or unmodified (corresponding to either WT ZIKV E or mutant ZIKV E) Emergent BioSolutions, Inc. plasmids (see Plasmids for details) using 8 µg of PEI per well. Twenty-four hours after transfection, fresh cell media was added to wells. Cells and cell media were collected 48 hours after transfection. Cell media was clarified by centrifugation for 3 minutes at 1500 RPM and then VLPs were lysed with 10X Triton buffer for a final dilution of 1X lysis buffer. Cells were collected with cold PBS, centrifuged for 3 minutes at 1500 RPM, and then lysed with Triton buffer. Cells and cell media were subjected to quantitative western blotting for the detection of ZIKV E by way of 4G2 antibody (concentration of 1:10,000). To calculate efficiency of VLP release, each ZIKV E protein value found in cells was first normalized to actin values in cells (rabbit anti-actin antibody at 1:5000). VLP release efficiency was then calculated as the quotient of the E protein quantity in VLPs divided by the quantity of E in cells [(E_mutant_VLP / E_mutant_cells) / (E_WT_ VLP/ E_WT_ cells)*100].

### Intracellular Infectious Virus Titer Assay and Authentic Virus Release Efficiency Calculation

To determine if there were differences in the intracellular titers of Y61 non-phosphorylatable mutant viruses compared to WT, distilled water was used to release intracellular virions from infected cells. To establish if there were differences in the release efficiencies of mutants compared to rWT virus, intracellular titers were compared to their corresponding extracellular titers and then normalized to rWT virus. The following methodology was adapted from Kong *et al.* [49]. First, 1.4 x 10^6^ Vero E6 cells were infected with ZIKV rWT, rY61V, or rY61F virus at an MOI of 0.001. After 96 hours, supernatants were clarified by centrifugation at 1500 RPM for 3 minutes to remove cells. Infected cells were centrifuged at 1500 RPM for 3 minutes then washed with PBS, 3 times in tandem, to remove any extracellular virions present in the supernatant. Washed cells were lysed with ultrapure distilled water for 15 minutes at room temperature. Then 2X DMEM was added to the lysate to normalize salts. Next, the lysate was centrifuged to separate cell debris from the intracellular virions in the supernatant. Finally, supernatants and lysates from infected cells were assayed for extracellular and intracellular titers, respectively, by plaque assay. Release efficiency was calculated using the following formula: [(Mutant _extracellular titer_ / Mutant_intracellular titer_) / (WT virus_extracellular titer_ / WTvirus_intracellular titer_)*100].

### Cell viability assay

To determine if rY61 mutant viruses impact cell viability, we used an MTT (3-(4,5-dimethylthiazol-2-yl)-2,5-diphenyltetrazolium bromide)) assay (M6494, Thermo Fisher Scientific). First, 1.2 x 10^5^ Vero E6 cells were infected with ZIKV rWT, rY61V, or rY61F virus. At 24, 48, and 96 hours post-infection, supernatants were collected, clarified by centrifugation at 1500 RPM for 3 minutes, stored at -80°C, then titered by plaque assay. At every collection time point, cells were treated with 1.2 mM MTT in PBS for 4 hours at 37°C. After incubation, MTT was removed and DMSO was added to each well, incubated for 10 minutes at 37°C, and absorbance was read at 570 nm. Cell viability was determined by normalizing the absorbance readings of mutant virus-infected wells to the rWT-infected control wells.

### Kinase inhibition assays

To determine if treating cells at the time of infection with various kinase inhibitors would impact viral growth, viral challenge assays were performed. First, to screen a panel of inhibitors at a fixed concentration, 2.5 x 10^5^ A549 cells were seeded in 6-well plates. The next day cells were infected at an MOI of 0.1 with ZIKV. After an hour of absorption at 37°C, the inoculum was removed and cells were treated with 10 µM of Sunitinib (B1045, APExBIO Technology LLC, Houston, TX) Bosutinib (PZ0192, Sigma-Aldrich), Defactinib (B4800, APExBIO), Imatinib (9084S, Cell Signaling Technology), or DMSO vehicle control in HEK293T media. The infection proceeded for 48 hours then supernatants were collected, clarified by centrifugation, stored at -80°C, and then titered by plaque assay. The percent inhibition was determined by normalizing titers of drug-treated wells to the DMSO control well titers and multiplying by 100. Second, to determine the IC_50_ of Bosutinib or Defactinib, a dose response curve was set up with drugs at concentrations of 30, 20, 15, 10, 7.5, 5, and 1.25 µM. 2.8 x 10^4^ A549 cells were seeded into 48 well plates. One day later, cells were infected at an MOI of 0.1. One hour after absorption, the inoculum was removed and cells were treated with A549 media containing drug at the listed concentrations or a DMSO vehicle control. After 48 hours, supernatants were collected, clarified by centrifugation, stored at -80°C, and then titered by plaque assay. To determine the cell toxicity of each drug, an MTT assay was conducted in parallel to the infection and kinase inhibitor treatment according to manufacturer instructions. A549 cells were set up in 48 well plates and treated with the listed concentrations of Bosutinib, Defactinib, or DMSO. After 48 hours, cell media was removed and replaced with 1.2 mM MTT in PBS for 4 hours at 37°C. After incubation, MTT was removed and DMSO was added to each well, incubated for 10 minutes at 37°C, and absorbance was read at 540 nm. Cell viability was determined by first normalizing the absorbance readings of drug-treated cells to the DMSO control wells. Cell toxicity was determined by subtracting the value of cell viability from 100. To determine the IC_50_ (half-maximal inhibitory concentration) of Bosutinib and Defactinib, we used [inhibitor] versus normalized response curve fitting in Prism 10.4. To determine CC_50_ (cytotoxic concentration causing death to 50% of cells), the same Prism 10.4 curve fitting was applied to our MTT assay data (inhibitor versus cell toxicity). The selectivity index (SI) of each drug was calculated by dividing the CC_50_ by the IC_50_ of each drug.

To determine if Bosutinib impacts growth of rY61 mutants, first, 3 x 10^5^ A549 cells were infected with ZIKV rWT, rY61V, or rY61F virus at an MOI of 0.01. After an hour of absorption at 37°C, the inoculum was removed and replaced with A549 media containing 15 µM Bosutinib or DMSO. After 48 hours, supernatants were collected, clarified by centrifugation, stored at -80°C, then titered by plaque assay.

## Supporting information

S1 Fig Identification of ZIKV E phosphosite T351 in virions from mosquito cells. C6/36 mosquito cells were inoculated with ZIKV at an MOI of 0.1. Four days later, cell-free ZIKV particles were collected, concentrated, and purified by banding on a potassium tartrate density gradient. (A) Aliquots of gradient fractions were separated by SDS-PAGE and stained with Coomassie Brilliant Blue; the position of ZIKV E is shown. Virus-containing fractions then were dialyzed and concentrated. (B) Negative stain transmission electron micrograph showing concentrated ZIKV virions prepared using this process (from an infection at MOI of 1.0, harvested at 72 h post-infection). Concentrated ZIKV particles were digested with trypsin and LysC and analyzed using mass spectrometry. Annotated mass spectra are shown for ZIKV E T351 in (C) unmodified site and (D) phosphorylated forms; ph/@ = phosphorylation.
(TIF)

S2 Fig Purification of plasmid-expressed ZIKV M, C, or E in the setting of phosphatase inhibition for mass spectrometry analysis. Western blots and Coomassie stained SDS-page gels of purified SBP-tagged ZIKV proteins. HEK293T cells were transfected with a plasmid encoding the indicated SBP-tagged ZIKV protein or, as a control, an empty vector (V). Two days after transfection, cells were treated with DMSO, H_2_O_2_, or Calyculin A for 20 minutes and then lysed in Triton-X buffer. SBP-tagged ZIKV proteins were affinity purified using streptavidin-coated magnetic beads and the captured protein mixtures were orthogonally separated using SDS-PAGE, followed by in-gel digestion for subsequent mass spectrometry analysis to discover PTMs or interacting host proteins. The molecular weight in kDa, viral proteins (yellow text and arrows), and immunoglobulin bands are labeled. Note that the asterisks in indicate monomeric streptavidin that was eluted off of the streptavidin beads when boiled.
(TIF)

S3 Fig Purification of plasmid-expressed ZIKV NS1, NS4A, and NS2B in the setting of phosphatase inhibition for mass spectrometry analysis. Western blots and Coomassie stained SDS-page gels of purified SBP-tagged ZIKV proteins. HEK293T cells were transfected with a plasmid encoding the indicated SBP-tagged ZIKV protein or, as a control, an empty vector (V). Two days after transfection, cells were treated with DMSO, H_2_O_2_, or Calyculin A for 20 minutes and then lysed in Triton-X buffer. SBP-tagged ZIKV proteins were affinity purified using streptavidin-coated magnetic beads and the captured protein mixtures were orthogonally separated using SDS-PAGE, followed by in-gel digestion for subsequent mass spectrometry analysis to discover PTMs or interacting host proteins. The molecular weight in kDa, viral proteins (yellow text and arrows), and immunoglobulin bands are labeled. Note that the asterisks in indicate monomeric streptavidin that was eluted off streptavidin beads when boiled.
(TIF)

S4 Fig Purification of plasmid expressed ZIKV NS4B and NS5 in the setting of phosphatase inhibition for mass spectrometry analysis. Western blots and Coomassie stained SDS-page gels of purified SBP-tagged ZIKV proteins. HEK293T cells were transfected with a plasmid encoding the indicated SBP-tagged ZIKV protein or, as a control, an empty vector (V). Two days after transfection, cells were treated with DMSO, H_2_O_2_, or Calyculin A for 20 minutes and then lysed in Triton-X buffer. SBP-tagged ZIKV proteins were affinity purified using streptavidin-coated magnetic beads and the captured protein mixtures were orthogonally separated using SDS-PAGE, followed by in-gel digestion for subsequent mass spectrometry analysis to discover PTMs or interacting host proteins. The molecular weight in kDa, viral proteins (yellow text and arrows), and immunoglobulin bands are labeled. Note that the asterisks in indicate monomeric streptavidin that was eluted off of the streptavidin beads when boiled.
(TIF)

S5 Fig Post-translational modifications labeled on an electrostatic model of ZIKV E dimer (PDB ID: 5JHM). Blue indicates positive charges while red indicates negative charges. (A) Electrostatic view of ZIKV E dimer. (B) rotated to the negatively charged “face”. (C) “Side-view” positioning showing the neutral border of electrostatically labeled ZIKV E dimer along with location of PTM site Y61.
(TIF)

S6 Fig ZIKV E Y61 interactions with neighboring residues and a view of the junction of ZIKV E and M. (A) Zoomed in view of the junction of ZIKV E (yellow) and M heterodimer (dark purple) with M phosphosites labeled in green. (B) Zoomed in view of Y61 on ZIKV E. Y61 is labeled in green while residues in close proximity, N207 and E262, are labeled in yellow.
(TIF)

S7 Fig NS2A and NS3 did not express at sufficiently high levels for mass spectrometry analysis. Attempts were made to express and purify NS2A and NS3 via plasmid transfection of mammalian cells. HEK293T cells were transfected with plasmids that encoded ZIKV proteins with either a C-terminal 3x FLAG-tag (A) or a SBP tag (B). Two days later, cells were lysed and tag-based protein purifications were attempted with an anti-FLAG antibody and magnetic protein G beads (A) or magnetic streptavidin beads (B).
(TIF)

S8 Fig Expression of WT and mutant forms of plasmid-derived ZIKV E. Accompanying Western blots of the VLP release assay shown in Fig 3F. HEK293T were transfected with a plasmid that encodes ZIKV prM and E (either WT E or the indicated E mutants). Forty-eight hours after transfection, cells and cell media (labeled “VLPs”) were subjected to quantitative western blotting for the detection of ZIKV E by way of 4G2 antibody or β-Actin. Note that for Biological Replicate 3, Technical Replicate 2, the Y61F and Y61E lanes in the VLP blot were in a different order compared to the rest of the biological and technical replicates shown. The samples actually loaded at each position for these 2 mutants on that particular blot are indicated.
(TIF)

S9 Fig Tyrosine 61 on the ZIKV E protein is critical for viral propagation at the stage of viral release. (A) The intracellular and extracellular titers of ZIKV rWT, rY61V, and rY61F were measured by focus assay. Vero E6 cells were infected with the indicated virus at an MOI of 0.5 for 24 hours. Supernatants were collected, infected cells were lysed with distilled (Milli-Q) water to release intracellular virions, as described in Materials and Methods, and titers were determined by focus assay. Data represent the mean±SEM from three independent biological replicates. Statistical significance was determined in Prism 10.4 software using a one-way ANOVA with Holm-Sidak’s test for multiple comparisons. (B) Release efficiency of authentic virions was calculated by normalizing the ratio of rY61 mutant extracellular to intracellular infectious titers (shown in panel A) to the ratio of rWT extracellular to intracellular infectious titers, as described in the Materials and Methods. Statistical significance was determined in Prism 10.4 software using a one-way ANOVA with Holm-Sidak’s test for multiple comparisons For the statistical tests in (A) and (B): *, P < 0.05; **; P < 0.01; ***; P < 0.001; ****; P < 0.0001.
(TIF)

S10 Fig Elevated titers of non-phosphorylatable rY61 mutant viruses correspond with higher cell viability. Left Y-axis: Vero E6 cells were infected with rWT, rY61V, or rY61F virus at an MOI of 0.001. Supernatants were collected at 24, 72, and 96 hours post-infection and viral titers (open bars) were determined by plaque assay. Data represent the mean±SEM from two biological replicates for the 24- and 96-hour time points, and mean±SEM from three biological replicates for the 72-hour time point. Right Y-axis: simultaneous to titer determination, cell viability (shaded bars) was measured by MTT assays at 24, 72, and 96 hours post-infection; see also Fig 3I. Data represent the mean±SEM from three biological replicates. Statistical significance was determined in Prism 10.4 software via two-way ANOVA with Holm-Sidak’s test for multiple comparisons. For the statistical test: *, P < 0.05; **; P < 0.01; ***; P < 0.001.
(TIF)

S11 Fig ZIKV-human kinase interaction network. The complete list of 115 interacting kinases found in this study (S2 Table) was subjected to MiST scoring with a score threshold of 0.62, resulting in 40 kinases and 85 viral-host interactions (S3 Table). The network depicts interactions between ZIKV proteins (purple circles / with orange border if ZIKV protein was phosphorylated) and cellular kinases (grey circles) discovered during mass spectrometry. Kinases involved in neurological disorders such as microcephaly, intellectual disability, developmental delays, vision and hearing issues, paralysis, and dementia (grey circle with red text), and kinases that were independently found in other ZIKV interactome or functional studies (grey circle with dashed borders) are indicated. Interactions between viral and host proteins are depicted by solid lines with color representing MiST scoring (lowest to highest corresponding to lightest to darkest purple, respectively) and width representing spectral counts (lowest to highest corresponding to thinnest to thickest, respectively). Note that S3 Table provides all interactions between a viral protein and host kinase, including those visually shown here, providing an alternate method to determine specific virus protein-host kinase interactions. Dashed lines indicate published interactions or associations between kinases integrated with the STRING application in Cytoscape 3.9.1.
(TIF)

S1 Data All tryptic peptides detected from mass spectrometry analysis of purified ZIKV virions in main Fig 1D. (XLSX)

S2 Data Zika virus protein peptides and post-translational modifications (PTMs) discovered with mass spectrometry. PTM notations are as follows: @, phosphorylation on S, T, or Y residues; ~ , ubiquitination at K residues; ^ carboxyamidomethylation on C residues; #, acrylamidation on C residues; and finally *, oxidation on M residues. The treatment conditions under which peptides were discovered are DMSO, H_2_O_2_, and Calyculin A and are color codes as yellow, cream, and dark yellow, respectively. ZIKV E peptides from infected cells were untreated.
(XLSX)

S3 Data The tandem mass spectra of discovered post translational modifications on ZIKV proteins. Tandem mass spectra were collected in a Q Exactive Plus mass spectrometer. Each spectrum is labeled with the peptide and ZIKV protein where a modification was found, as well as the residue number on the ZIKV protein. Spectra of residues are shown first without post-translational modifications then the same residues labeled with “p” or “ub” to represent phosphorylation or ubiquitination, respectively. Special characters within peptide sequences indicate modifications as follows: @ = phosphorylation, * = oxidation, ~ = ubiquitination, ^ = carbamidomethylation, # = acrylamidation. Accompanying tables show the corresponding calculated and measured b- and y-type ions (colored in blue and red, respectively) indicating identified fragment ion masses.
(PDF)

S4 Data A multiple sequence alignment of flavivirus sequences using MUSCLE algorithm. See ‘Multiple sequence alignment’ under Materials and Methods for additional details.
(PDF)

S1 Table Sequences of phosphorylated and ubiquitinated peptides discovered by mass spectrometry categorized by ZIKV protein. Phosphorylations are indicated by the phosphorylated residue followed by @. Ubiquitinations are indicated by the ubiquitinated residue followed by ~.
(XLSX)

S2 Table Complete list of cellular kinases that associate with ZIKV proteins. Columns A and B contain kinase IDs and full protein names, followed by gene name synonyms in Column C. Column D and E indicate the designated kinase group or family/subfamily, respectively. Column F indicates whether kinases were independently discovered in other ZIKV proteomics (Scaturro et al., Shah et al., Coyaud et al.) or functional screens (Savidis et al., Li et al., Wang et al.). Column G lists the ZIKV protein(s) each kinase was found to interact with in this study; M, E, NS1, NS2B, NS4B are phosphoproteins. Column H contains the sum total spectral counts of kinase peptides discovered by mass spectrometry in all conditions (DMSO, Calyculin A, and H_2_O_2_) corresponding with the respective, in-line ZIKV proteins in Column G. Column I indicates whether kinases are predicted by Scansite 4.0 to phosphorylate the listed ZIKV proteins at discovered phosphosites (See Table 1). Column J lists inhibitors known to target indicated kinases. Column K lists the MiST score of the host protein and viral protein interaction in each line or row.
(XLSX)

S3 Table Cellular kinases that associate with ZIKV proteins that score above the MiST threshold of 0.62. Columns A, B, C contain kinase IDs, full protein names, and gene name synonyms, respectively. Column D and E indicate the designated kinase group or family/subfamily, respectively. Column F indicates whether kinases were independently discovered in other ZIKV proteomics (Scaturro et al, Shah et al., Coyaud et al.) or functional screens (Savidis et al., Wang et al.). Columns G lists which ZIKV protein(s) kinases were found to interact with in this study; M, E, NS1, NS2B, NS4B are phoshoproteins. Column H contains the sum total spectral counts of kinase peptides discovered by mass spectrometry in all conditions (DMSO, Calyculin A, and H_2_O_2_) corresponding with the respective, in-line ZIKV proteins in Column G. Column I indicates whether kinases are predicted by Scansite 4.0 to phosphorylate the listed ZIKV proteins at discovered phosphosites (See Table 1). Column J lists inhibitors known to target indicated kinases. Column K lists the MiST score of the host protein and viral protein interaction in each line or row.
(XLSX)

S4 Table Primers used for Gateway subcloning of ZIKV genes (C, E, M, NS1, NS2B, or NS4A) with a 3XFLAG tag from the pOME plasmid to a modified pCAGGS expression vector. Each protein was engineered to express from the pCAGGS vector with a C-terminal streptravidin-binding petpide (SBP). Note the 3XFLAG tag was not included in the pCAGGS vector. Additional details described in Materials and Methods under ‘Plasmids.’
(XLSX)
