# Supplementary material for: A global map of the Zika virus phosphoproteome reveals host-driven regulation of viral particle release and cytopathogenicity
Source: PLoS Pathog. 2026 Sep 3;22(9):e1014300. doi: 10.1371/journal.ppat.1014300 (PMC13541192; doi:10.1371/journal.ppat.1014300)

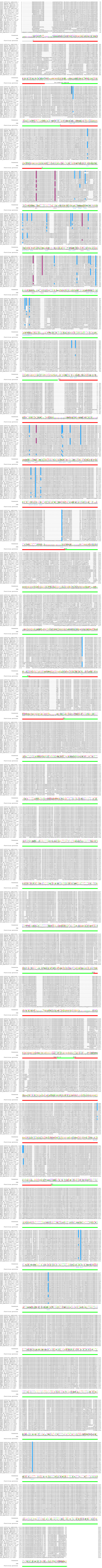

Supplement: Supplementary file 2 [file ppat.1014300.s015.pdf]
